# CSF1R and IL1R1 inhibitors synergistically attenuate the early pathogenesis of traumatic brain injury in mice

**DOI:** 10.1016/j.neurot.2025.e00787

**Published:** 2025-11-07

**Authors:** Sudena Wang, Yong Wang, Jenny Strehle, Isa Wernersbach, Ermis Papakonstantinou, Pawit Somnuke, Katharina Ritter, Matthias Klein, Irmgard Tegeder, Michael K.E. Schäfer

**Affiliations:** a Department of Anesthesiology, University Medical Center, Johannes Gutenberg-University Mainz, Langenbeckstr. 1, 55131, Mainz, Germany; b Department of Plastic Surgery, Renmin Hospital of Wuhan University, Wuhan, 430060, Hubei Province, China; c Department of Anesthesiology, Faculty of Medicine Siriraj Hospital, Mahidol University, Bangkok, 10700, Thailand; d Institute for Immunology, University Medical Center, Johannes Gutenberg-University Mainz, Langenbeckstr. 1, 55131, Mainz, Germany; e Focus Program Translational Neurosciences (FTN) of the Johannes Gutenberg-University Mainz, Langenbeckstr. 1, 55131, Mainz, Germany; f Institute of Clinical Pharmacology, Goethe-University Frankfurt, Medical Faculty, Theodor Stern Kai 7, 60590, Frankfurt, Germany; g Research Center for Immunotherapy (FZI), Johannes Gutenberg-University Mainz, Langenbeckstr. 1, 55131, Mainz, Germany

**Keywords:** Traumatic brain injury, CSF1R, IL1R1, Neuroinflammation, Therapy

## Abstract

There is an unmet need in the treatment of traumatic brain injury (TBI), a leading cause of death and disability. Colony stimulating factor 1 receptor (CSF1R) and interleukin 1 receptor type 1 (IL1R1) are critical regulators of TBI-associated neuroinflammation. This study tested the hypothesis that early administration of CSF1R inhibitor PLX3397 plus IL1R1 inhibitor Anakinra alleviates TBI pathogenesis. Adult C57BL/6 mice were subjected to experimental TBI and treated with PLX3397 plus Anakinra, PLX3397 or Anakinra alone, or vehicle for up to five days post injury (5 dpi). Neurological deficits were attenuated by PLX3397 plus Anakinra in male and female mice. Combination therapy, as opposed to monotherapy, also reduced structural brain damage; however, this effect was observed exclusively in male mice. Bulk RNA-sequencing analysis of differentially expressed genes (DEGs) and gene set enrichment analysis (GSEA) revealed anti-neuroinflammatory effects in male mice treated with PLX3397 plus Anakinra, which exceeded the summed effects of monotherapies. Key DEGs included pro-neuroinflammatory markers such as *Cd68* and *Spp1*/osteopontin, as well as genes associated with type I and II interferon responses. Immunofluorescence staining confirmed that PLX3397 plus Anakinra was more effective than monotherapy in attenuating CD68^+^ macrophages/microglia, CD45^+^/CD68^-^ leukocytes, and osteopontin. Again, these effects exceeded the summed effects of monotherapy. The findings demonstrate beneficial synergistic effects of FDA-approved CSF1R and IL1R1 inhibitors and offer novel insights into the mechanisms of early TBI pathogenesis and therapy in a clinically relevant model.

## Graphical abstract

**Figure:**
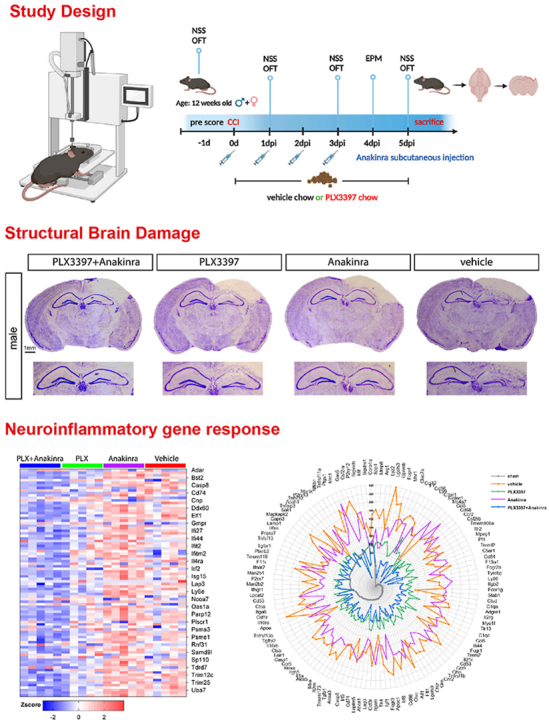

## Introduction

Traumatic brain injury (TBI) is a major cause of death and disability, presenting a significant medical and socioeconomic burden [1]. Current therapeutic options are mostly limited to surgical interventions and symptomatic relief, and only modest effects of various pharmacological treatments have been reported in recent randomized clinical trials [[2], [3], [4], [5]]. TBI pathology combines multiple pathophysiological processes, including cerebral ischemia, blood brain barrier (BBB) disruption, brain edema, mitochondrial dysfunction, oxidative stress, neural cell death, and inflammation [[6], [7], [8], [9]]. Microglia are considered the major drivers of the inflammatory responses, exerting, however, dual effects on the progression of damage following TBI [[10], [11], [12]].

Novel perspectives in investigating microglial function in health and disease emerged through the utilization of pharmacological inhibitors (i.e. PLX3397 and PLX5622) of colony stimulating factor 1 receptor (CSF1R) [[13], [14], [15], [16]]. CSF1R is a transmembrane receptor tyrosine kinase that is expressed in microglia and other myeloid lineage cells. Two endogenous ligands, CSF1/M-CSF and IL-34, have been identified that initiate downstream signaling cascades to regulate proliferation, differentiation and survival [17]. Although rigorous microglia depletion strategies are unlikely to be an adequate translational therapy for TBI [16,18], transient administration of CSF1R inhibitors at acute, subacute, or chronic phases of TBI resulted in beneficial effects [[19], [20], [21]]. Recently, we showed that administering PLX3397 during the first five days post-TBI reduced macrophage/microglia accumulation in injured brain tissue by 50 ​%. Discontinuing treatment at 5 dpi led to long-term beneficial effects at 30 dpi, which were more pronounced in male than in female mice [21]. However, this therapeutic approach was not sufficient to reduce the structural brain damage at 5 dpi, suggesting that CSF1R-independent pathomechanisms were involved and may have impeded therapeutic improvement. Indeed, the observation that gene expression of both IL-1β and IL1R1 remained unaltered, despite substantial reduction of macrophages/microglia, indicated a crucial role of IL1R1 signaling by other cell types in this context [21].

IL1R1 is a receptor for IL-1α, IL-1β, and IL-1ra, primarily activating NF-κB and MAPK pathways, and the expression of key inflammatory mediators [22]. IL1-IL1R1 signaling has been implicated in the activation of innate immunity following TBI, including the production of pro-inflammatory cytokines, recruitment of leukocytes and monocytes, disruption of BBB integrity, and exacerbation of neural cell death [[23], [24], [25], [26], [27], [28]]. Anakinra, is the first IL-1ra drug that competitively prevents association of IL-1α, IL-1β with IL-1R1 [29]. It is approved as Kineret® for treatment of rheumatoid arthritis [30], Still’s disease including systemic juvenile idiopathic arthritis and adult-onset Still’s disease [31], cytokine storm syndromes [32], and COVID-19 disease [33]. Moreover, its safety was demonstrated in a randomized clinical phase II trial in TBI patients, but the study lacked sufficient statistical power to assess patient outcomes [34]. In rodent models of TBI, Anakinra has been shown to provide overall beneficial effects on the inflammatory response, neurological deficits, and structural brain damage [[35], [36], [37], [38]].

Hence, CSF1R and IL1R1 play a pivotal role in inflammatory processes and are promising therapeutic targets in TBI. While CSF1R is predominantly expressed by microglia [39], IL1R1 is also expressed in various other cell types, including neurons, astrocytes, and endothelial cells [24], suggesting that therapeutic approaches targeting these pathways simultaneously may enhance the therapeutic efficacy following TBI. Therefore, we tested the hypothesis that a combination treatment consisting in PLX3397 plus Anakinra improves the outcome of TBI at the behavioral, histological and gene expression level. We subjected cohorts of sex-matched adult C57BL/6JRj mice to the controlled cortical impact (CCI) model of TBI and compared 4 treatment groups i.e. PLX3397 plus Anakinra, PLX3397, Anakinra, vehicle. Mice were monitored for neurological deficits for up to five days post injury (5 dpi), followed by the examination of brain structural damage, transcriptomic changes, and brain tissue inflammation at 5 dpi.

## Material and Methods

### Animals and TBI experiments

Adult, 12-14 week-old male and female C57BL/6JRj mice (Janvier Germany) were housed and maintained in a controlled environment with a 12-h dark/light cycle, temperature of 22 ​± ​1 ​°C, and humidity of 50 ​± ​5 ​%. Food and water were available to them *ad libitum*. All experiments were conducted in accordance with the institutional guidelines of the Johannes Gutenberg University, Mainz, Germany, and were approved by the Animal Care and Ethics Committee of the Landesuntersuchungsamt Rheinland-Pfalz (protocol number G19-1-027) and followed the ARRIVE guidelines. Mice were treated in TBI/sham experiments essentially as described [40]. Anesthesia was induced with 4 ​% isoflurane for 90 ​s and maintained with 2.1 ​% isoflurane throughout the procedure. For analgesia, mice received carprofen (Rymadyl®, Zoetis) which was injected 30 ​min before start. After achieving the desired level of anesthesia, lidocaine-HCl (2 ​%, B.Braun) was injected subcutaneously at the temples, and the mouse head was then fixed in a stereotactic frame (Kopf Instruments). The temperature was maintained at 37 ​°C using feedback heating (Hugo Sachs, MarchHugstetten, Germany). The skin was incised 1 ​cm along the midline and a craniotomy of 4 ​× ​4 ​mm was drilled above the right parietal cortex. The bone fragment was flapped laterally while ensuring that the dura mater remained intact. The cortical impact was induced using a Benchmark™ Stereotaxic Impactor (Leica Biosystems, Wetzlar, Germany) with the following parameters: impactor tip diameter of 3 ​mm, velocity of 6 ​m/s, duration of 200 ​ms, and displacement of 1.5 ​mm. After achieving hemostasis, the skull bone fragment was repositioned into the burr hole and sealed with tissue glue, followed by the suturing of the skin incision. Sham mice received isoflurane anesthesia, underwent skin incision and suturing, but did not undergo craniotomy or impact injury. For recovery, mice were placed in a temperature (36 ​°C) controlled incubator (IC8000, Draeger, Luebeck, Germany) and transferred back to their home cages after 90 ​min. Two animals died during the CCI procedure and were replaced. All animals survived the post-traumatic observation period until 5 dpi.

### Experimental design and pharmacological treatment

**Fig. 1:**
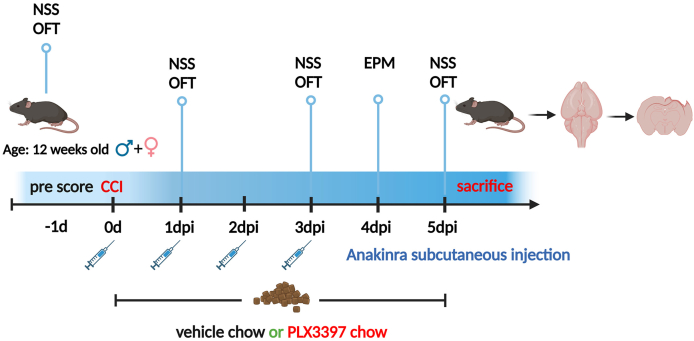
**Experimental design and pharmacological treatment**. NSS and OFT were conducted at 1 day before TBI (-1d), and subsequently at 1, 3 and 5 dpi. EPM was performed at 4 dpi. Mice (equal sex ratio) were administered PLX3397 (290 ​mg/kg body weight) or vehicle chow for a duration of 5 days. Anakinra (100 ​mg/kg body weight) or placebo was injected subcutaneously at 5 ​min, 1, 2, and 3 days following CCI surgery. At 5 dpi, brains were dissected and processed for histological analysis and RNAseq using cryosectioning. This figure was generated using Biorender.

A total of 56 mice were randomly divided into 5 experimental groups with equal sex distribution: TBI ​+ ​PLX3397+Anakinra (*n* ​= ​12), TBI ​+ ​PLX3397 (*n* ​= ​12), TBI ​+ ​Anakinra (*n* ​= ​12), TBI ​+ ​vehicle (*n* ​= ​12), and sham ​+ ​vehicle (*n* ​= ​8) (Fig. 1). Mice received either PLX3397 or vehicle chow (Research Diets) *ad libitum*, beginning 90 ​min after the CCI/sham procedure until the study’s endpoint at 5 dpi. Anakinra (100 ​mg/kg, Kineret, Amgen) or placebo (saline) was injected subcutaneously at 5 ​min, and 1, 2, and 3 dpi. Previous studies demonstrated the effectiveness of dietary PLX3397 administration at a food pellet concentration of 290 ​mg/kg [13,21]. Daily food consumption was determined from 1 dpi to 5 dpi, and body weights were recorded at 1, 3, and 5 dpi (Figs. S1 and S2 supplementary material). The researchers who performed the surgeries, drug administration, behavioral testing, data collection, and analyses were blinded to the group identities.

### Evaluation of the neurological severity score (NSS) and behavioral monitoring

To evaluate neurological deficits, behavioral monitoring was performed in the same order as the CCI/sham operations between 9:00 a.m. and 2:00 p.m. Neurological impairments were assessed using the NSS, which measures balance, motor function, and general behavior, one day before TBI and at 1, 3, and 5 dpi essentially as described [41]. The NSS scores from 0 (no impairment) to 12 (severe neurological dysfunction), and pre-injury scores were close to zero, as expected. The general locomotor activity of the mice was evaluated in an open field test (OFT) (40 ​× ​40 ​× ​40 ​cm, 330–350 Lux) using a video tracking system (EthoVisionXT, Noldus, Wageningen, Netherlands) before and after TBI (pre-score, 1, 3, and 5 dpi). Mice were positioned in the center of the arena, their movements was recorded for 3 ​min, and the moving distance was calculated using the EthoVisionXT14 software. The elevated plus maze (EPM) test was utilized to assess anxiety/impulsivity at 4 dpi. A standard EPM maze 50 ​cm above ground with two closed arms with opaque walls and two open arms without walls was used. Mice were initially placed into the central area facing an open arm. Their movement was monitored and recorded continuously for 5 ​min. Using EthoVisionXT14 software, the duration spent in open and closed arms and the frequency of entries into each arm were analyzed.

### Brain sectioning and histopathology

The processing of brain tissue was carried out as described [42]. Mice were subjected to deep anesthesia using 4 ​vol% isoflurane and subsequently decapitated at 5 dpi. Brains were carefully extracted, rapidly frozen in powdered dry ice, and stored at −20 ​°C. A cryotome (HM 560 Cryo-Star, Thermo Fisher Scientific, Walldorf, Germany) was used to cut coronal brain sections, 12 ​μm thick, across 16 consecutive levels at 500 ​μm intervals from Bregma +3.14 ​mm to −4.36 ​mm, which were collected on Superfrost Plus Adhesion Microscope slides (New Erie Scientific LLC, USA). Intermediate sections (60 ​μm, 8 ​× ​4), which were divided along the midline to separate the right and left hemispheres, were obtained from Bregma +0.64 ​mm to −2.86 ​mm. The upper quadrants of sections containing lesioned and perilesional brain (tissue including the cortex, striatum, dorsal hippocampus, and thalamus) were frozen in liquid nitrogen and processed for RNA extraction and RNA sequencing (RNAseq) as previously described in detail [21].

Cresyl violet staining was performed to evaluate the structural brain damage as described [42]. Briefly, brain sections underwent air-drying at room temperature (RT) for 1 ​h, followed by rehydration in 70 ​% ethanol for 2 ​min, staining in cresyl violet solution (10 ​mg/ml, 20 ​% ethanol, Merck) for 10 ​min, rinsing with distilled water, and dehydration using increasing concentrations of ethanol (70 ​%, 96 ​%, and 100 ​%). Hematoxylin-Eosin (H&E) staining was applied to evaluate the area of hematoma as described [21].

Immunofluorescence staining was performed with cryosections, which were air-dried at RT for 30 ​min. The sections were then fixed in 4 ​% paraformaldehyde for 10 ​min, followed by washing in phosphate-buffered saline (1x PBS). A blocking solution consisting of 5 ​% goat serum, 0.5 ​% bovine serum albumin, and 0.1 ​% Triton X-100 in 1x PBS was applied to the sections at RT for 1 ​h. Primary antibodies were added to the blocking solution and incubated overnight at 4 ​°C. The next day, the sections were washed with 1x PBS and incubated with secondary antibodies in the blocking solution for 1.5 ​h at RT. After washing in 1x PBS, the sections were mounted in Immu-Mount (Fisher Scientific). Primary and secondary antibodies and dilutions are listed as supplementary material (Table S1).

### Image acquisition and histopathological analyses

Sections stained with cresyl violet or H&E were visualized using a bright field microscope (Stemi 305, Zeiss). Quantification of lesion volume, hippocampal granule cell layer (GCL) thickness, and Cornu ammonis 1 (CA1) length was conducted using Zen software (RRID: SCR_013672), and the hematoma area was determined utilizing FIJI ImageJ software (RRID: SCR_003070) [21]. Briefly, the volume of brain lesions was determined by identifying regions lacking violet staining in the injured hemisphere across 16 consecutive brain (Bregma +3.14 ​mm to −4.36 ​mm) cryosections and multiplying the area by the distance between two sections (500 ​μm). The relative lesion volume was calculated by dividing the lesion volume by the total ipsilateral hemisphere volume [43]. GCL thickness was evaluated at 5 dpi in the dorsal hippocampus from Bregma levels −2.36 ​mm to −2.86 ​mm on two sections along the suprapyramidal blade [41]. The CA1 length was determined on two sections from Bregma levels −2.36 ​mm to −2.86 ​mm. Values of GCL thickness are expressed as % relative to the contra-lesional hemisphere, and values of CA1 length are expressed as % of preserved CA1 length. Determination of hematoma size was performed with H&E-stained sections (three per animal) from Bregma levels −0.36 ​mm to −1.36 ​mm. Immunofluorescence was acquired with a fluorescence microscope (BZ-X800, Keyence) on three sections from Bregma levels −0.36 ​mm to −1.36 ​mm by investigators blinded to the experimental randomization using equal acquisition parameters for each staining combination. Quantitative assessments were performed with FIJI ImageJ (RRID: SCR_003070) using auto-threshold settings implemented in FIJI followed by the “analyze particle” plugin.

### RNAseq and analysis

Brain sample total RNA was extracted from cryosections containing damaged and surrounding areas (including cortex, striatum, dorsal hippocampus, and thalamus) as previously described in detail [21]. Briefly, total RNA was purified using RNAeasy Kit (Qiagen), and the quantity and quality assessment was checked by Qubit 2.0 and RNA 6000 Nano chip on Agilent’s bioanalyzer, respectively. Next Generation paired-end RNAseq was performed at Novogene (Cambridge, UK) on an Illumina NovaSeq 6000 platform. Sample quality was evaluated using demultiplexed fastq.gz files, and sequenced reads were subjected to adapter trimming and processing via CLC Genomics workbench (CLC, Qiagen, v21.0.5). Results were visualized using CLC expression browser, encompassing the number of mapped reads, target length, source length and position, strand, genes and gene IDs, annotated according to the mm10 assembly. Read counts were normalized and the expression value unit is TPM (in CLC called CPM). Principal component analysis (PCA, Fig. S3) was performed with CLC using Z-score normalization across samples for each gene, genes with zero expression across all samples are removed. Data are deposited GSE228309 (see availability of data and materials).

The results were displayed as Volcano plots (absolute difference >4 between compared groups), VENN diagrams (https://bioinformatics.psb.ugent.be/webtools/Venn/) and gene set enrichment analyses (GSEA) (http://www.gsea-msigdb.org) [44] to assess functional implications of up- or downregulated genes. Differential gene expressions were calculated using ANOVA-like methods, t-tests and fold change, with p values ​< ​0.05, as detailed the figure legends.

### Statistical analysis

GraphPad Prism software (GraphPad Software, version 10.0) was used to analyze the data. Shapiro-Wilk normality test was used to assess the data distribution. Outliers were detected using the Rout’s test, excluded from subsequent analyses, as specified in the figure legends. To compare different treatment groups, parametric data were analyzed using one-way analysis of variance (ANOVA) or Brown-Forsythe and Welch ANOVA tests, depending on the SD variance (F test), while nonparametric data were assessed using the Kruskal‒Wallis test. Two-way ANOVA or mixed effects model, with subsequent Holm-Šídák post hoc test was used to analyze differences between groups in behavioral testing for the between subject factor “treatment” and the within subject factor “time”. Post hoc analyses for parametric and nonparametric data were conducted using the Holm-Šídák, Dunnett T3, or Dunn’s multiple comparison tests, respectively. The sample size was calculated assuming that a 20 ​% change in the analyzed criteria would be relevant. The Type I error probability was set at *α* ​= ​0.05, while the standard power was set at 1 ​− ​*β* ​= ​0.8 (80 ​%), which results in a Type II error probability of *β* ​= ​0.2. The values for individual animals are shown as the mean ​± ​standard error of the mean (SEM). *p* ​< ​0.05 was considered to indicate statistical significance; ∗*p* ​< ​0.05, ∗∗*p* ​< ​0.01, ∗∗∗*p* ​< ​0.001, ∗∗∗∗*p* ​< ​0.0001, ns ​= ​not significant. All p values and F values are reported in the supplementary information (Tables S4-S8).

## Results

### PLX3397 plus Anakinra therapy reduces neurological deficits after TBI

**Fig. 2:**
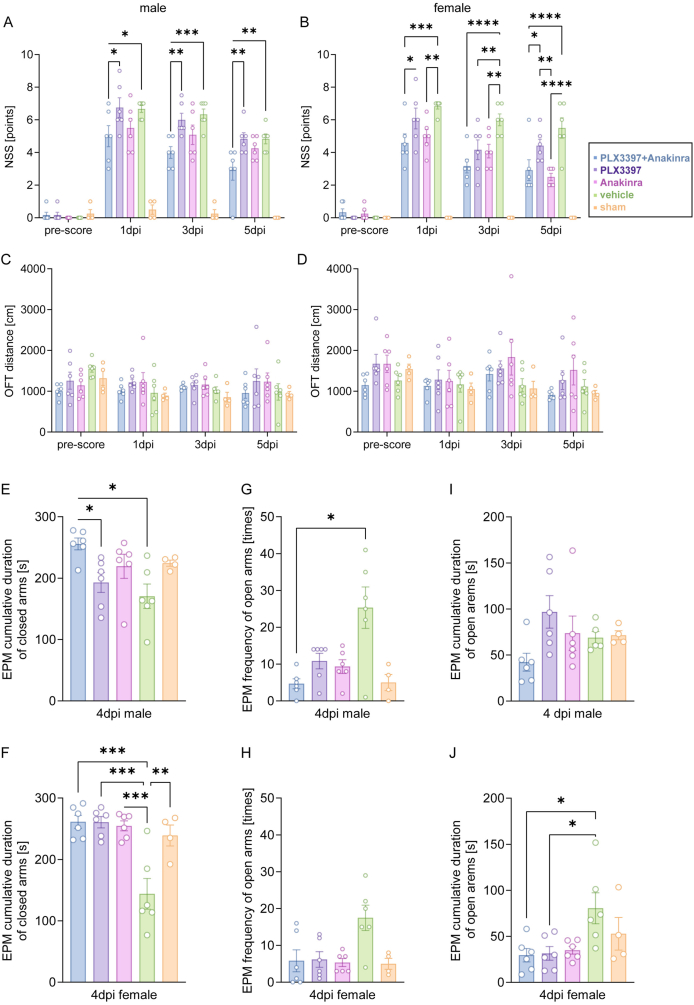
**Combined PLX3397 plus Anakinra therapy reduces neurological deficits after TBI.** (A, B) NSS in male and female mice at 1 day before TBI (pre-score), 1, 3, and 5 dpi. Higher scores indicate greater neurological severity. (C, D) OFT in male and female mice at one day before TBI, 1, 3, and 5 dpi. Longer distances indicate increased locomotor activity. (E–J) EPM in male and female mice at 4 dpi. (E, F) Cumulative time spent in closed arms. Higher latency indicates increased anxiety-related behavior. (G, H) Frequency of open arm entries. Increased frequency indicates impaired anxiety-related behavior. (I, J) Cumulative time spent in open arms. Higher latency indicates impaired anxiety-related behavior. Data points represent individual mice, TBI: *n* ​= ​6 per group, sham: *n* ​= ​4 per group, data are expressed as mean ​± ​SEM. Two outliers (B: sham, I: TBI vehicle) were identified by Rout’s test and excluded. Two-Way ANOVA with mixed-effects model followed by Holm-Šídák multiple comparisons test (A–D). One-way ANOVA and Kruskal‒Wallis test followed by post hoc Holm-Šidák or Dunn’s corrections (E–J), ∗*p* ​< ​0.05, ∗∗*p* ​< ​0.01, ∗∗∗*p* ​< ​0.001, ∗∗∗∗*p* ​< ​0.0001.

TBI was induced by CCI in adult sex-matched C57BL/6JRj mice and drug treatment started on the day of surgery. While the CSF1R inhibitor PLX3397 was administered via drug-containing chow (290 ​mg/kg) until 5 dpi, treatment with the IL1R1 inhibitor Anakinra was performed using four subcutaneously injections (100 ​mg/kg each), shortly after TBI, at 1, 2, and 3 dpi (Fig. 1). Motor function deficits, anxiety-like and open-field locomotion were assessed (Fig. 2A–J). NSS, with higher scores indicating increased severity, was assessed at 1, 3, and 5 dpi and was consistently lower in both male and female mice receiving the combination therapy of PLX3397 plus Anakinra versus vehicle (Fig. 2A and B). Monotherapy with Anakinra showed similar effects, albeit more pronounced in female mice than in male mice, whereas mice treated with PLX3397 alone had no advantage over vehicle, irrespective of sex (Fig. 2A and B). Locomotor activity of male and female mice in the OFT at 1, 3, and 5 dpi did not differ between CCI-treatment groups (Fig. 2C and D). However, mice treated with vehicle or monotherapy tended to show more TBI-evoked hyperactivity than mice receiving PLX3397 plus Anakinra (Fig. 2C and D). In EPM at 4 dpi, vehicle treated CCI mice spent less time in the closed arms and showed a higher frequency of visits to the open arm as compared to pharmacologic treatments or sham but differences were only significant for the combination PLX3397 plus Anakinra vs. vehicle for both sexes (Fig. 2E–H). The total time in the open arm summed over all time points was not different between treatment groups of male mice (Fig. 2I). However, in female mice, the total open arm time was significantly reduced, almost reaching sham level, when mice were treated with either PLX3397 plus Anakinra or single PLX3397 (Fig. 2J). Overall, the results showed that combined therapy reduced neurological deficits irrespective of sex and was superior to monotherapy with PLX3397. However, Anakinra showed also some behavioral effects in female mice. Combined therapy almost normalized exploratory and anxiety-related behavior compared to vehicle, albeit not consistently for all parameters. The superior therapeutic efficacy of the PLX3397+Anakinra was not accompanied with adverse effects at the behavioral level.

### PLX3397 and Anakinra increase hematoma size at 5 dpi

**Fig. 3:**
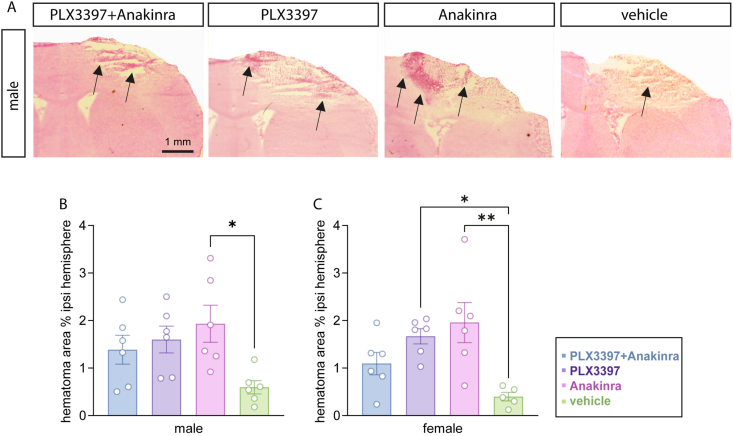
**PLX3397 and Anakinra increase hematoma size at 5 dpi.** (A) H&E staining showing intracerebral hematoma (arrows point to hematoma) in the ipsilesional brain tissue in male mice with TBI (scale: 1 ​mm, Bregma −0.86 ​mm). (B, C) Relative hematoma area (% ipsilesional hemisphere) is increased following Anakinra vs. vehicle treatment in male mice and following PLX3397 and Anakinra monotherapy vs. vehicle in female mice. Data points represent individual mice (*n* ​= ​6 per group), data are expressed as mean ​± ​SEM. One outlier (C: TBI vehicle) was identified by Rout’s test and excluded. One-way ANOVA followed by post hoc Holm-Šidák, ∗*p* ​< ​0.05, ∗∗*p* ​< ​0.01.

Intracerebral hemorrhages and the subsequent formation of hematoma are pathological hallmarks of TBI, and consistently occur with the CCI model of TBI [45]. The removal of the hematoma largely depends on microglial activity [21]. Hematomas were visualized by H&E staining at 5 dpi (Fig. 3A). They were confined to the ipsilesional hemisphere, and their relative size was calculated across three Bregma levels in each animal. A trend toward increased mean hematoma size was observed for all drug treatment groups compared to vehicle both in male and in female mice (Fig. 3B and C). However, hematoma size did not significantly increase in PLX3397 plus Anakinra treated mice, whereas statistically significant effects were observed following Anakinra monotherapy in male and in female mice, and PLX3397 monotherapy in female mice (Fig. 3B and C).

### PLX3397 plus Anakinra therapy attenuates structural brain damage in male mice

**Fig. 4:**
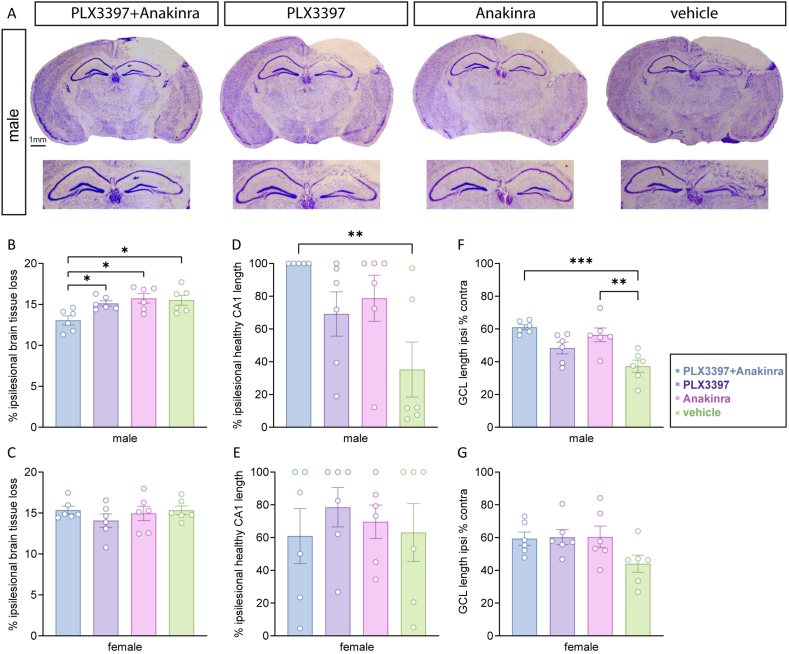
**PLX3397 plus Anakinra attenuates structural brain damage in male mice.** (A) Cresyl violet staining showing coronal brain sections from male mice at 5 dpi (scale: 1 ​mm, Bregma −2.36 ​mm). (B, C) Relative brain tissue loss (% of ipsilesional hemisphere) is attenuated in PLX3397+Anakinra vs. monotherapy or vehicle exclusively in male mice. (D, E) Relative ipsilesional healthy CA1 length (% of total ipsilesional CA1 length) is preserved in PLX3397+Anakinra compared to vehicle exclusively in male mice. (F, G) Relative ipsilesional GCL length (% of contra GCL length) is preserved following combined PLX3397+Anakinra therapy and Anakinra monotherapy compared to vehicle in male mice. Data points represent individual mice (*n* ​= ​6 per group), data are expressed as mean ​± ​SEM. One outlier (D: TBI PLX3397+Anakinra) was identified by Rout’s test and excluded. One-way ANOVA and Kruskal‒Wallis test followed by post hoc Holm-Šidák or Dunn’s corrections, ∗*p* ​< ​0.05, ∗∗*p* ​< ​0.01, ∗∗∗*p* ​< ​0.001.

To examine effects of combined PLX3397 plus Anakinra therapy on structural brain damage, brain lesion size as well as structural integrity of the hippocampal CA1 region and GCL were analyzed in cresyl violet-stained sections (Fig. 4A). Brain lesion volumes were calculated across sixteen Bregma levels (+3.14 ​mm to −4.36 ​mm) at 5 dpi. Combined PLX3397 plus Anakinra therapy resulted in smaller lesion volumes in male mice as compared to all other TBI groups (Fig. 4B). Monotherapy using Anakinra or PLX3397 did not affect brain lesion size as compared to vehicle. Interestingly, the neuron-preserving therapeutic effect of combined PLX3397 plus Anakinra was evident only in male mice and not in female mice (Fig. 4B and C). We next assessed the degree of CA1 region preservation at 5 dpi, which is located beneath the directly injured cortex (Fig. 4D and E). The mean preservation rate of the CA1 region in vehicle treated male mice was about 35.26 ​% (±16.78 ​%, SEM), whereas it was almost 100 ​% (near total preservation) in male mice treated with PLX3397 plus Anakinra (one outlier with 41.9 ​% was recognized as an outlier). Monotherapies with PLX3397 or Anakinra reached preservation rates of 69.19 ​% (±13.53 ​%, SEM) and 78.78 ​% (±14.04 ​%, SEM), respectively, without reaching a statistically significant difference versus vehicle treated male mice (Fig. 4D). In female mice, CA1 preservation rates ranging from 60 to 78 ​% were reached, but without differences between treatment groups (Fig. 4E). Similarly, preservation of the hippocampal GCL was improved after combined PLX3397 plus Anakinra therapy vs. vehicle in male mice, and Anakinra monotherapy also showed protective effects for GCL integrity as compared to vehicle in male mice (Fig. 4F). In female mice, both combined PLX3397 plus Anakinra and monotherapies with Anakinra or PLX3397 mildly increased GCL preservation but without reaching statistical significance (Fig. 4G). Taken together, PLX3397 plus Anakinra reduced structural brain damage and was superior to monotherapy at 5 dpi in male mice. However, female mice did exhibit significant benefits from single administration of PLX3397, Anakinra, or their combination, versus vehicle treatment.

### Transcriptomic profiling demonstrates synergistic efficacy of PLX3397 plus Anakinra therapy

Male mice had a stronger benefit in brain tissue protection than female mice, this led us to focus on male animals in subsequent analyses. To characterize the beneficial effects of combined PLX3397 plus Anakinra at the molecular level, we employed RNAseq of brain samples collected at 5 dpi. Principal component analysis showed clustering of treatment groups. Overlapping clustering was observed between PLX3397+Anakinra and PLX3397 monotherapy on the one hand, and Anakinra monotherapy and TBI vehicle on the other hand, all of which were clearly separated from sham mice. In addition, treatment-specific effects on transcriptomic profiles were evident from gene heatmaps including significantly regulated genes and Top 100 regulated genes (Figs. S3-S5).

**Fig. 5:**
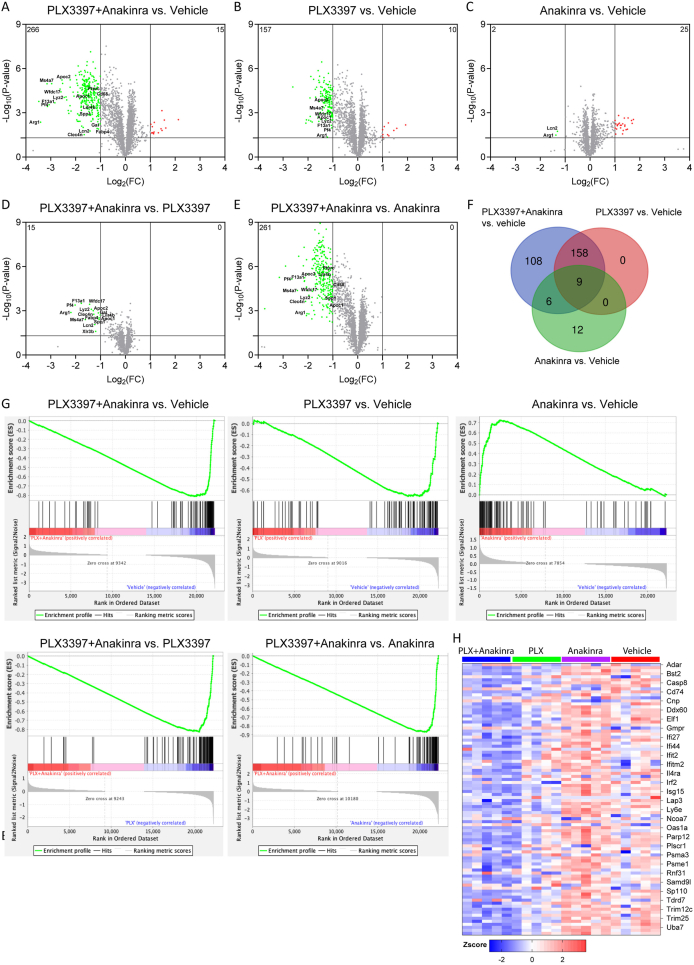
**Transcriptomic profiling demonstrates synergistic efficacy of PLX3397 plus Anakinra therapy.** (A–E) Volcano plots show DEGs (criterion: absolute difference >4, fold change >2, *p* ​< ​0.05) in PLX3397+Anakinra and monotherapy versus vehicle, as well as PLX3397+Anakinra versus monotherapy (*n* ​= ​5 per group). The X-axis represents the log2-transformed fold change (log2 FC) and the Y-axis represents the negative log10-transformed *p*-value. Significantly up- or down-regulated DEGs (red dots ​= ​upregulated, green dots ​= ​downregulated) and non-significantly regulated genes (gray dots) are indicated. (F) VENN diagram of DEGs in PLX3397+Anakinra, PLX3397, and Anakinra treatment groups versus vehicle. (G) Enrichment plots, generated using GSEA 4.1.0, show the hallmark “IFNα response” related gene sets in PLX3397+Anakinra and monotherapy versus vehicle, as well as PLX3397+Anakinra versus monotherapy. The green line chart represents the gene enrichment score (ES) and the peak represents maximum enrichment. The vertical black bars represent individual genes of the gene set. The bottom part shows the change in all genes, red indicates high expression and blue indicates low expression. (H) Heatmap showing relative expression levels (Zscores) of 90 genes belonging to hallmark “IFNα response”, red to blue color indicates high to low expression.

Next, DEGs between the different groups were calculated and visualized as volcano scatter plots (Fig. 5A–E, Fig. S6, supplementary data). The number of DEGs was obviously higher in PLX3397 plus Anakinra versus vehicle as compared to PLX3397 versus vehicle, and DEGs were scarce for Anakinra versus vehicle (Fig. 5A–C). Hence, the comparisons between TBI groups versus vehicle showed an increasing number of downregulated DEGs in PLX3397 plus Anakinra ​≫ ​PLX3397 ​> ​Anakinra. Comparisons between PLX3397+Anakinra versus monotherapy further revealed DEGs exclusively downregulated with the combination versus PLX3397 (15 genes) and the combination versus Anakinra (261 genes) (Fig. 5D and E). This analysis suggested unique transcriptomic effects of PLX3397+Anakinra therapy.

Accordingly, a VENN diagram revealed unique and shared up- or downregulated DEGs between treatment groups and vehicle, and demonstrated marked differences in the number of DEGs (PLX3397 plus Anakinra versus vehicle: 281 genes; PLX3397 versus vehicle: 167 genes; Anakinra versus vehicle: 27 genes, Fig. 5F, Tables S2.1 – S32.5, supplementary data). This analysis further confirmed more pronounced transcriptomic changes following combination therapy than with either PLX3397 or Anakinra monotherapy, and also versus the summed monotherapy-DEGs (167 ​+ ​27 ​< ​281). Notably, 108 DEGs were exclusively found in the comparison PLX3397 plus Anakinra versus vehicle. There were no exclusive DEGs for PLX3397 because all “PLX3397-dependent” DEGs were shared with the combination. Comparison of Anakinra monotherapy and vehicle showed 12 DEGs exclusively regulated by Anakinra (Table S2.1 – S2.5, supplementary data).

STRING-pathway analysis was used to identify gene ontology (GO) terms and pathways associated with DEGs identified by volcano plots and those identified by the VENN diagram (Tables S2 and S3, supplementary data). Both types of analysis showed that the majority of DEGs for PLX3397 plus Anakinra versus vehicle or monotherapy, were downregulated and associated with GO terms related to immune system processes and immune response. In contrast, Anakinra versus vehicle revealed more up-regulated DEGs, which were associated with ECM organization and collagen synthesis (Tables S2.1 – S2.5, supplementary data).

Furthermore, these effects were also evident when calculating the number of DEGs between all TBI groups vs. sham mice. Combined PLX3397 plus Anakinra therapy reduced strikingly the number of up-regulated DEGs in response to TBI vehicle by 44 ​% (from 856 to 478 DEGs). PLX3397 reduced the number of up-regulated DEGs by 28 ​% (from 856 to 620 DEGs) whereas Anakinra rather increased the number of DEGs by 3.4 ​% (from 856 to 885 DEGs) (Fig. S6, supplementary data).

GSEA was used to calculate enrichments based on all annotated transcripts, providing an expanded overview of GO associations of BP, MF and CC as well as hallmark genes [44]. We examined GO associations (GOBP, GOMF, and GOCC) and hallmark gene sets. Again, among the significantly downregulated GO terms in mice with PLX3397 plus Anakinra in comparison to mice treated with vehicle, PLX3397 or Anakinra monotherapy were predominantly associated with immune and inflammation processes (Fig. S7, supplementary data).

Notably, enrichment plots and heatmaps for “interferon alpha response (IFNα)” or “interferon gamma (IFNγ) response” clearly showed the downregulation of IFN-I/II response hallmark genes in PLX3397 plus Anakinra versus all other treatment groups (Fig. 5G, H, Fig. S8, supplementary data). In contrast, Anakinra alone versus vehicle associated with up-regulation of both IFN-I/II response hallmark genes (Fig. 5G, H, Fig. S8, supplementary data).

While down-regulated DEGs supported anti-inflammatory effects of PLX3397 plus Anakinra, we also examined GO associations of up-regulated DEGs. Neuronal-, neurotransmission-, and synapse-associated GO terms were increased for mice receiving the combination of PLX3397 plus Anakinra versus mice treated with vehicle, PLX3397 or Anakinra monotherapy (Fig. S9, supplementary data).

**Fig. 6:**
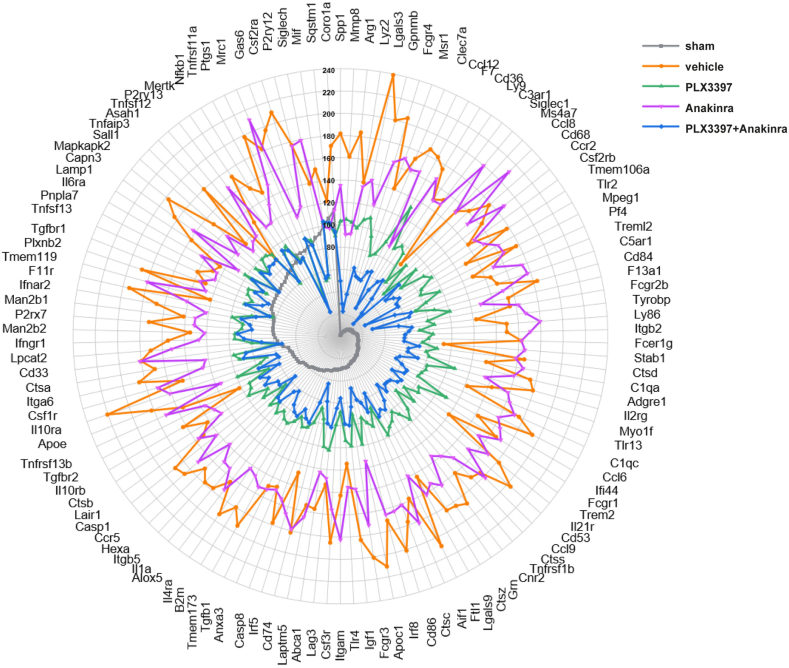
**PLX3397 plus Anakinra down-regulates microglia-associated genes.** Radar plot showing averaged gene expression of individual microglia-associated DEGs (identified by text mining using 10x Genomics CellMarker 2.0, http://117.50.127.228/CellMarker/index.html) in TBI treatment groups and sham. Expression values are relative to the overall average (= common mean) of individual genes. The strongest increase across TBI groups was found for Lgals3 in vehicle TBI (about 240 ​% of the common mean).

Given the crucial role of microglia as a therapeutic target and in the inflammatory response to TBI, we screened for microglia-associated genes (out of a total of 4546 DEGs, as calculated by ANOVA) significantly regulated in our model of TBI using text mining (10x Genomics CellMarker 2.0, https://117.50.127.228/CellMarker/index.html). We identified 126 microglia-associated genes (Fig. 6) and their expression was strongly reduced with PLX3397 monotherapy and even stronger with the combination PLX3397 plus Anakinra versus TBI vehicle. This analysis revealed that an inhibited microglial response to TBI strongly contributed to the therapeutic efficacy of PLX3397 plus Anakinra.

### PLX3397 plus Anakinra therapy attenuates brain tissue infiltration by activated microglia and leukocytes

**Fig. 7:**
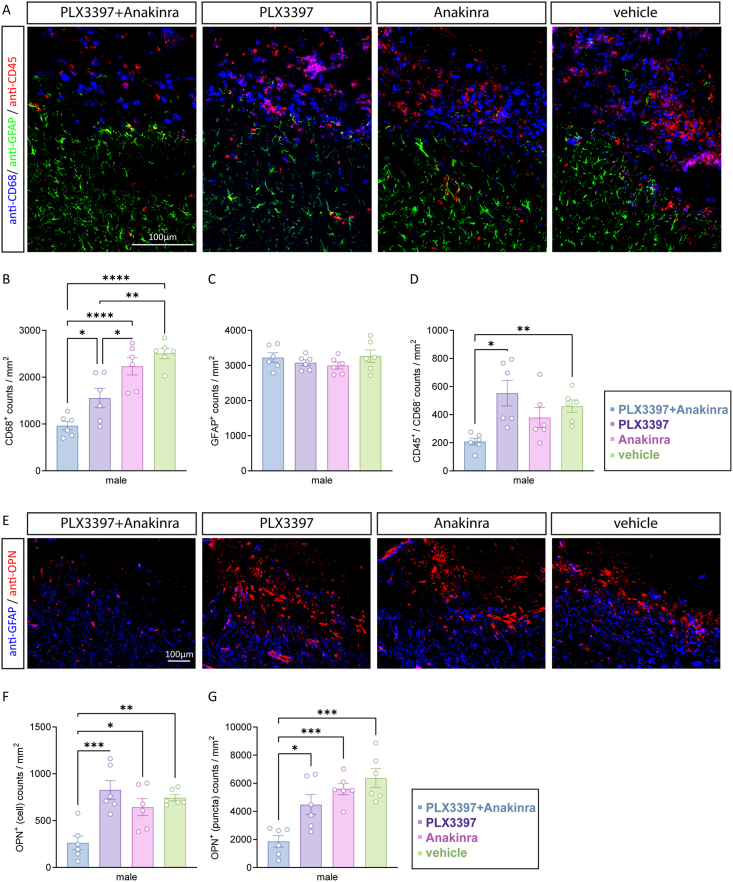
**PLX3397 plus Anakinra attenuates CD68^+^, CD45^+^ and osteopontin**^+^**cells brain tissue infiltration in male mice.** (A) Triple immunostaining of the ipsilesional cortex (scale: 100 ​μm, Bregma −0.86 ​mm) using anti-CD68/CD45/GFAP antibodies showing activation of microglia, leukocytes and astrocytes. (B–D) Column plots showing CD68^+^, GFAP^+^, and CD45^+^/CD68^-^ cell counts in mice treated with PLX3397+Anakinra compared to PLX3397 monotherapy, Anakinra monotherapy or vehicle. (E) Representative images of immunostained ipsilesional cortex (scale: 100 ​μm, Bregma −0.86 ​mm) using anti-osteopontin showing TBI-induced accumulation of OPN^+^ cells and OPN^+^ puncta. Sections were counterstained with anti-GFAP to identify perilesional brain regions. (F, G) Column plots showing reduced OPN^+^ cell counts and OPN^+^ puncta counts in PLX3397+Anakinra compared to monotherapy or vehicle mice. Data points represent individual mice (*n* ​= ​6 per group), data are expressed as mean ​± ​SEM. One-way ANOVA and Brown-Forsythe ANOVA test followed by post hoc Holm-Šidák or Dunnett T3 corrections, ∗*p* ​< ​0.05, ∗∗*p* ​< ​0.01, ∗∗∗*p* ​< ​0.001, ∗∗∗∗*p* ​< ​0.0001.

Among the down-regulated microglia-associated genes following PLX3397+Anakinra therapy were *Cd68* and *Spp1* (encoding for osteopontin, OPN). These genes were recently reported to be significantly up-regulated in our model of TBI at 5 dpi [42]. In addition, *Ptprc* (encoding for CD45), a pan-marker of leukocytes was significantly down-regulated in the PLX3397+Anakinra versus vehicle group (Fig. 5A). Brain sections from male mice at 5 dpi were immunostained with antibodies specific to CD68, OPN, GFAP or CD45 to simultaneously examine, macrophages/microglia, astrocytes, and peripheral immune cells in the injured brain tissue (Fig. 7). First, immunofluorescence revealed robust activation of CD68^+^ microglia and GFAP^+^ astrocytes at perilesional sites, and their numbers were clearly increased following TBI compared to sham animals, as expected (Fig. S10, supplementary data). While the numbers of astrocytes were not affected by our treatment approach, mice treated with PLX3397+Anakinra exhibited lower numbers of CD68^+^ microglia compared to those from monotherapy or vehicle treated mice in the perilesional brain tissue (Fig. 7A–C). Furthermore, we observed diminished numbers of CD45^+^/CD68^-^ leukocytes after combined PLX3397 plus Anakinra compared to PLX3397 or vehicle treatment (Fig. 7A–D). In addition, cell counts of OPN^+^ cells revealed a clear reduction at perilesional sites in the PLX3397 plus Anakinra group versus monotherapy and vehicle (Fig. 7E and F). The same effect was observed for small OPN^+^ puncta, likely resembling a pool of soluble OPN deposited in the injured brain tissue (Fig. 7E–G).

## Discussion

Previous results from our laboratory have shown that early post-traumatic treatment with the CSF1R inhibitor PLX3397 attenuated the proliferation and accumulation of microglia/macrophages by approximately 50 ​% at 5 days after experimental TBI, but without reduction of the structural brain damage at this time point [21]. Considering that potentially neurotoxic pathways such as IL1-IL1R1 signaling are also mediated by other cell types, we now investigated whether combined inhibition of CSF1R plus IL1R1 provided additional therapeutic benefit. Indeed, the results show that the combination of PLX3397 plus Anakinra was superior to either treatment alone and improved the outcome compared with vehicle at the behavioral, morphologic and molecular level. The only aspect that remained beyond therapeutic reach was the hematoma. However, compared to monotherapies, which tended to increase hematoma volume, the combination of PLX3397 plus Anakinra was more similar to vehicle, indicating no worsening occurred. [46,47]. Nevertheless, these observations should be approached with caution, as hematomas are not evenly distributed across the injured brain, and we assessed hematoma size in a limited number of sections. To further explore this issue, a more comprehensive study is warranted, using for example various MRI modalities to quantify hematomas, alongside cerebral edema, which offers predictive value for injury severity and functional outcome in animal models of TBI [47,48].

Overall, male mice had a stronger benefit in brain tissue protection than female mice. This led us to focus on male animals in subsequent analyses, including RNAseq and immunofluorescence analyses. Indeed, sex-specific differences in the response to CSF1R inhibition by PLX3397 have been recently reported with male mice showing a more pronounced depletion of microglia following a two- or three-week treatment with PLX3397 [49,50]. Body weight and food consumption had little influence on the distribution of PLX3397 in these studies, as brain concentrations were similar in male and female mice [49,50]. In contrast, long-term treatment over two to twenty-two months resulted in higher PLX3397 concentrations in the brain of male mice compared to female mice in a model of Alzheimer’s Disease [51]. While we found little evidence in a previous study for sex-specific PLX3397 effects after 5 days of treatment post TBI, PLX3397 decreased expression of few microglial marker in male, but not in female sham mice [21]. To our knowledge, sex-specific effects of Anakinra in mice have not been reported, and this was also not notably observed in the present study. The sex-specific effects in our study may be related to an overall stronger neuroinflammatory response in male versus female mice in the CCI model of TBI, which also correlated with the size of brain lesions [52]. Sex steroid hormones are thought to be at least partially responsible for such effects, since both pharmacological manipulation of their synthesis or ovariectomy affects microglial and astrocyte activation and other pathogenic processes during the acute phase in the CCI model of TBI [53,54].

Interestingly, sexual dimorphism has been associated with the early activation of microglia and infiltration of pro-inflammatory myeloid cells during the initial days following brain injury in male mice, but not in female mice [55]. Since IL-1/IL1R1 signaling facilitates the *trans*-endothelial migration of leukocytes, including myeloid cells [56,57], and CSF1R signaling regulates proliferation and survival of microglia in acute TBI [21], it is conceivable that similar mechanisms account for the here observed stronger therapeutic responsiveness of male mice in our study. Along this line, infiltrating leukocytes have been shown to be involved in the neuroinflammatory response in models of acute traumatic or ischemic brain injuries and indicated a role in the amplification of inflammatory signaling via crosstalk with brain resident cells, particularly with microglia [[58], [59], [60], [61], [62], [63], [64]]. In addition, IL1-IL1R1 signaling both in endothelial cells and astrocytes may affect leukocyte recruitment and inflammatory amplification [56,65,66]. Furthermore, PLX3397 and Anakinra are also inhibitors of systemic immune responses [13,[67], [68], [69]]. Systemic effects are likely contributing to the results of our study, as the attenuation of TBI-induced systemic inflammatory responses may also reduce brain infiltration. This reduction may, in turn, decrease the mutual stimulation of leukocytes, microglia, and potentially other cell types. Consequently, it will be important to assess the role of systemic immunosuppression, in addition to the drug effects observed in the brain tissue, in future studies.

Our results showing superior efficiency of combined treatment with PLX3397 plus Anakinra versus single drug in reducing structural brain damage, CD68^+^ macrophages/microglia, and CD45^+^/CD68^-^ leukocytes at injury sites suggested a synergistic mode of action of PLX3397 and Anakinra. We performed bulk RNAseq, GO enrichment analysis of DEGs and GSEA to test this hypothesis. Indeed, the transcriptomic response to the PLX3397 plus Anakinra, represented as the number of DEGs, exceeded the sum of the responses to monotherapy with PLX3397 or Anakinra. DEGs in the PLX3397 plus Anakinra versus vehicle or versus monotherapy groups were mostly down-regulated and associated with GOBP terms like “immune response” (GO:0006955), “regulation of immune system process” (GO:0002682) and “cytokine production” (GO:0001816). Moreover, GSEA revealed opposing drug effects on the IFN-I/II responses. While PLX3397 treatment inhibited this response, Anakinra led to its upregulation. For the combination of PLX3397 and Anakinra, the PLX3397-dependent downregulation predominated and the Anakinra-only effect was overruled. In fact, the combination therapy more effectively reduced the IFN-I/II responses than PLX3397 alone, although the individual drugs had opposing effects. This suggests that the outcome with the combination treatment is not a simple additive effect but rather a synergistic neuroprotective and anti-inflammatory mechanism. Therefore, we propose that synergistic brain tissue protection by PLX3397 plus Anakinra is, at least in part, attributable to a reduction of the pathologic interaction between leukocytes and microglia and the associated amplification of inflammatory signaling.

This hypothesis is supported by several findings in models of TBI. For example, infiltrating monocytes/macrophages have been demonstrated to influence the activation of microglia, thereby promoting IFN-I responses and neuroprotective effects were observed when this microglial response was reduced [70]. Furthermore, signaling via cyclic GMP-AMP-synthase-Stimulator of Interferon Genes (cGAS-STING) has been demonstrated to regulate early IFN-I-driven inflammatory and detrimental crosstalk of peripheral immune cell infiltration and microglia in TBI [71]. The significant role of the IFN-I pathway and its upstream regulation has been established across various experimental models of TBI, encompassing different age groups and post-traumatic time points [[72], [73], [74], [75], [76], [77], [78], [79], [80]]. The substantial evidence supporting the critical role of IFN-I signaling, combined with our findings that IFN-I responses contribute to the synergistic effects of PLX3397 plus Anakinra therapy, support the potential value of pharmacologically targeting this pathway in TBI.

It is important to acknowledge that the results of the present study are context-specific for the CCI model of TBI. While CCI is a widely used model, it does not fully replicate the complexity and heterogeneity of human TBI [81,82]. Furthermore, initiating PLX3397 and Anakinra administration shortly after TBI - with the first Anakinra injection given 5 ​min and PLX3397 administration with food after the TBI or sham procedure - does not reflect a clinical scenario. The focus on the early phase of TBI is another major limitation and our study does not provide predictions for the subacute and late outcomes which may depend on sex, age, injury model, brain regions, pre-injury conditions, and post-injury time [21,[83], [84], [85]]. It is evident that future studies need to investigate lasting benefits or treatment associated risks, which do not reveal within the first days of treatment. It will be necessary to evaluate the relevance of PLX3397 plus Anakinra treatment for cognitive outcomes, as clinical studies on TBI have found persistent cognitive deficits [86,87]. In rodent models, such deficits can be studied using a battery of behavioral tests suitable for assessing long-term cognitive and hippocampal-dependent abnormalities, such as spatial learning and memory, fear conditioning, and novel object recognition [[88], [89], [90]].

In addition, a recently published transcriptomic single-cell atlas demonstrated that microglia display the highest degree of heterogeneity across murine TBI models, sex, brain region, and time points [84]. These findings contribute to the existing body of research indicating that distinct brain regions exhibit varied responses to TBI due to differences in their proximity to the injury site, patterns of neuronal damage, and immune response [[91], [92], [93], [94], [95]]. For example, the hippocampal CA1 region, which was mostly preserved in our study following treatment with PLX3397 and Anakinra, is particularly vulnerable to pro-apoptotic processes induced by TBI (Tehranian et al., 2008). Therefore, the neuroprotective effects of PLX3397 plus Anakinra support a significant role of neuroinflammation-driven secondary brain injury progression in the CCI model of TBI, which may be amenable to immune-targeting treatments. As a next step, it will be crucial to evaluate treatment effects on the longitudinal and regional changes of microglial subpopulations in phenotypes and functions [[96], [97], [98]] as well as other neural and immune cell types. Recent integration of single cell data across two time points (1 and 7 dpi) and in two brain regions (cortex and hippocampus) has shed light on the complex cellular and molecular processes following TBI [94]. This spatiotemporal systems approach may prove particularly valuable in characterizing therapeutic effects.

Since we focused our RNAseq pathway analysis and immunofluorescence studies on male mice, we might have missed special beneficial effects of the combinatorial PLX3397 plus Anakinra treatment in female mice. Finally, we did not investigate in this study whether the use of PLX3397 and/or Anakinra, either as combined therapy or monotherapy, induces systemic immune suppression. It remains to be determined if such treatment would curtail the pathologic amplifying and detrimental interactions between leukocytes and microglia.

## Conclusion

The translation of experimental TBI and its treatments to the clinical setting is still limited, also including drug repurposing [99,100]. This study demonstrates that CSF1R and IL1R1 inhibitors synergistically mitigate early brain damage, neurological deficits, and neuroinflammation following TBI. Our findings suggest that PLX3397 (Pexidartinib) and Anakinra has a potential for drug repurposing in TBI and support the view that multi-target drug combinations are superior to single drug treatment [101,102]. However, further research is required to evaluate long-term effects, as well as to examine sex-specific responses in more detail and subtle side effects which were not monitored in the current study.

## Ethics approval

All experiments were conducted in accordance with the institutional guidelines of the Johannes Gutenberg University, Mainz, Germany, and were approved by the Animal Care and Ethics Committee of the Landesuntersuchungsamt Rheinland-Pfalz (protocol number G19-1-027) and followed the ARRIVE guidelines.

## Consent for publication

Not applicable.

## Availability of data and materials

Source data are available upon reasonable request. RNAseq data have been deposited as GEO dataset with the provisional accession number GSE228309. https://www.ncbi.nlm.nih.gov/geo/query/acc.cgi?acc=GSE228309.

The following secure token has been created to allow review of record GSE228309 while it remains in private status: ivefcakavncnjcl.

To review GEO accession GSE228309: Go to: https://emea01.safelinks.protection.outlook.com/?url=https%3A%2F%2Fwww.ncbi.nlm.nih.gov%2Fgeo%2Fquery%2Facc.cgi%3Facc%3DGSE228309&amp;data=05%7C02%7C%7C68235eea66954e7b977708dd85abbcf5%7C84df9e7fe9f640afb435aaaaaaaaaaaa%7C1%7C0%7C638813694837236335%7CUnknown%7CTWFpbGZsb3d8eyJFbXB0eU1hcGkiOnRydWUsIlYiOiIwLjAuMDAwMCIsIlAiOiJXaW4zMiIsIkFOIjoiTWFpbCIsIldUIjoyfQ%3D%3D%7C0%7C%7C%7C&amp;sdata=S786XcNntfcl5aIM0lfmOOO9fXfeMtAmOvYMB6GNEDs%3D&amp;reserved=0.

Enter token ivefcakavncnjcl into the box.

## Author contributions

S.W., Y.W., J.S. I.W., E.P., P.S., K.R., M.K., I.T. performed experiments and/or data analysis. J.S., K.R., I.T., and M.S. conceived study design, animal testing protocols, and experiments. S.W., Y.W., I.T. and M.S. wrote the manuscript. All authors approved the final manuscript.

## Funding

This study was supported by the Deutsche Forschungsgemeinschaft (SCHA1261/4 ​− ​3 to MKES and CRC1080, C02 to IT) and the Chinese Scholarship Council (to SW).

## Declaration of competing interests

The authors declare that they have no competing interests.

## References

[1] Dewan M.C., Rattani A., Gupta S., Baticulon R.E., Hung Y.C., Punchak M. (2018). Estimating the global incidence of traumatic brain injury. J Neurosurg.

[2] Ritter K., Somnuke P., Hu L., Griemert E.V., Schäfer M.K.E. (2024). Current state of neuroprotective therapy using antibiotics in human traumatic brain injury and animal models. BMC Neurosci.

[3] Syzdykbayev M., Kazymov M., Aubakirov M., Kurmangazina A., Kairkhanov E., Kazangapov R. (2024). A modern approach to the treatment of traumatic brain injury. Medicines.

[4] Wright D.W., Yeatts S.D., Silbergleit R., Palesch Y.Y., Hertzberg V.S., Frankel M. (2014). Very early administration of progesterone for acute traumatic brain injury. N Engl J Med.

[5] Nichol A., French C., Little L., Haddad S., Presneill J., Arabi Y. (2015). Erythropoietin in traumatic brain injury (EPO-TBI): a double-blind randomised controlled trial. Lancet.

[6] Werner C., Engelhard K. (2007). Pathophysiology of traumatic brain injury. Br J Anaesth.

[7] Schäfer M.K., Pfeiffer A., Jaeckel M., Pouya A., Dolga A.M., Methner A. (2014). Regulators of mitochondrial Ca(2+) homeostasis in cerebral ischemia. Cell Tissue Res.

[8] Akamatsu Y., Hanafy K.A. (2020). Cell death and recovery in traumatic brain injury. Neurotherapeutics.

[9] Abdul-Muneer P.M., Chandra N., Haorah J. (2015). Interactions of oxidative stress and neurovascular inflammation in the pathogenesis of traumatic brain injury. Mol Neurobiol.

[10] Donat C.K., Scott G., Gentleman S.M., Sastre M. (2017). Microglial activation in traumatic brain injury. Front Aging Neurosci.

[11] Morganti-Kossmann M.C., Semple B.D., Hellewell S.C., Bye N., Ziebell J.M. (2019). The complexity of neuroinflammation consequent to traumatic brain injury: from research evidence to potential treatments. Acta Neuropathol.

[12] Obukohwo O.M., Oreoluwa O.A., Andrew U.O., Williams U.E. (2024). Microglia-mediated neuroinflammation in traumatic brain injury: a review. Mol Biol Rep.

[13] Elmore M.R., Najafi A.R., Koike M.A., Dagher N.N., Spangenberg E.E., Rice R.A. (2014). Colony-stimulating factor 1 receptor signaling is necessary for microglia viability, unmasking a microglia progenitor cell in the adult brain. Neuron.

[14] Green K.N., Crapser J.D., Hohsfield L.A. (2020). To kill a microglia: a case for CSF1R inhibitors. Trends Immunol.

[15] Han J., Zhu K., Zhang X.M., Harris R.A. (2019). Enforced microglial depletion and repopulation as a promising strategy for the treatment of neurological disorders. Glia.

[16] Weyer M.P., Strehle J., Schäfer M.K.E., Tegeder I. (2024). Repurposing of pexidartinib for microglia depletion and renewal. Pharmacol Therapeut.

[17] Stanley E.R., Chitu V. (2014). CSF-1 receptor signaling in Myeloid cells. Cold Spring Harbor Perspect Biol.

[18] Boland R., Kokiko-Cochran O.N. (2024). Deplete and repeat: microglial CSF1R inhibition and traumatic brain injury. Front Cell Neurosci.

[19] Henry R.J., Ritzel R.M., Barrett J.P., Doran S.J., Jiao Y., Leach J.B. (2020). Microglial depletion with CSF1R inhibitor during chronic phase of experimental traumatic brain injury reduces neurodegeneration and neurological deficits. J Neurosci: Off J Soc Neurosci.

[20] Bray C.E., Witcher K.G., Adekunle-Adegbite D., Ouvina M., Witzel M., Hans E. (2022). Chronic cortical inflammation, cognitive impairment and immune reactivity associated with diffuse brain injury are ameliorated by forced turnover of microglia. J Neurosci: Off J Soc Neurosci.

[21] Wang Y., Wernersbach I., Strehle J., Li S., Appel D., Klein M. (2022). Early posttraumatic CSF1R inhibition via PLX3397 leads to time- and sex-dependent effects on inflammation and neuronal maintenance after traumatic brain injury in mice. Brain Behav Immun.

[22] Weber A., Wasiliew P., Kracht M. (2010). Interleukin-1 (IL-1) pathway. Sci Signal.

[23] Thome J.G., Reeder E.L., Collins S.M., Gopalan P., Robson M.J. (2019). Contributions of Interleukin-1 receptor signaling in traumatic brain injury. Front Behav Neurosci.

[24] Bodnar C.N., Watson J.B., Higgins E.K., Quan N., Bachstetter A.D. (2021). Inflammatory regulation of CNS barriers after traumatic brain injury: a tale directed by Interleukin-1. Front Immunol.

[25] Ozen I., Ruscher K., Nilsson R., Flygt J., Clausen F., Marklund N. (2020). Interleukin-1 beta neutralization attenuates traumatic brain injury-induced microglia activation and neuronal changes in the globus pallidus. Int J Mol Sci.

[26] Lin H.W., Basu A., Druckman C., Cicchese M., Krady J.K., Levison S.W. (2006). Astrogliosis is delayed in type 1 interleukin-1 receptor-null mice following a penetrating brain injury. J Neuroinflammation.

[27] Flygt J., Ruscher K., Norberg A., Mir A., Gram H., Clausen F. (2018). Neutralization of Interleukin-1β following diffuse traumatic brain injury in the mouse attenuates the loss of mature oligodendrocytes. J Neurotrauma.

[28] Lindblad C., Rostami E., Helmy A. (2023). Interleukin-1 receptor antagonist as therapy for traumatic brain injury. Neurotherapeutics.

[29] Arnold D.D., Yalamanoglu A., Boyman O. (2022). Systematic review of safety and efficacy of IL-1-Targeted biologics in treating immune-mediated disorders. Front Immunol.

[30] Fleischmann R.M., Schechtman J., Bennett R., Handel M.L., Burmester G.R., Tesser J. (2003). Anakinra, a recombinant human interleukin-1 receptor antagonist (r-metHuIL-1ra), in patients with rheumatoid arthritis: a large, international, multicenter, placebo-controlled trial. Arthritis Rheum.

[31] Maniscalco V., Abu-Rumeileh S., Mastrolia M.V., Marrani E., Maccora I., Pagnini I. (2020). The off-label use of anakinra in pediatric systemic autoinflammatory diseases. Ther Adv Musculoskelet Dis.

[32] Mehta P., Cron R.Q., Hartwell J., Manson J.J., Tattersall R.S. (2020). Silencing the cytokine storm: the use of intravenous anakinra in haemophagocytic lymphohistiocytosis or macrophage activation syndrome. Lancet Rheumatol.

[33] Mohamed Hussein A.A.R., Sayad R., Abdelshafi A., Hammam I.A., Kedwany A.M., Elkholy S.A. (2023). A meta analysis on the utility of Anakinra in severe COVID-19 disease. Cytokine.

[34] Helmy A., Guilfoyle M.R., Carpenter K.L., Pickard J.D., Menon D.K., Hutchinson P.J. (2014). Recombinant human interleukin-1 receptor antagonist in severe traumatic brain injury: a phase II randomized control trial. J Cerebr Blood Flow Metabol.

[35] Perez-Polo J.R., Rea H.C., Johnson K.M., Parsley M.A., Unabia G.C., Xu G.Y. (2016). Inflammatory cytokine receptor blockade in a rodent model of mild traumatic brain injury. J Neurosci Res.

[36] Newell E.A., Todd B.P., Mahoney J., Pieper A.A., Ferguson P.J., Bassuk A.G. (2018). Combined blockade of Interleukin-1α and -1β signaling protects mice from cognitive dysfunction after traumatic brain injury. eNeuro.

[37] Sun M., Brady R.D., Wright D.K., Kim H.A., Zhang S.R., Sobey C.G. (2017). Treatment with an interleukin-1 receptor antagonist mitigates neuroinflammation and brain damage after polytrauma. Brain Behav Immun.

[38] Evans L.P., Woll A.W., Wu S., Todd B.P., Hehr N., Hedberg-Buenz A. (2020). Modulation of post-traumatic immune response using the IL-1 receptor antagonist anakinra for improved visual outcomes. J Neurotrauma.

[39] Erblich B., Zhu L., Etgen A.M., Dobrenis K., Pollard J.W. (2011). Absence of colony stimulation factor-1 receptor results in loss of microglia, disrupted brain development and olfactory deficits. PLoS One.

[40] Ritter K., Jung K., Dolderer C., Appel D., Oswald C.C., Ritz U. (2021). Early reciprocal effects in a murine model of traumatic brain injury and femoral fracture. Mediat Inflamm.

[41] Hummel R., Ulbrich S., Appel D., Li S., Hirnet T., Zander S. (2020). Administration of all-trans retinoic acid after experimental traumatic brain injury is brain protective. Br J Pharmacol.

[42] Wang S., Weyer M.P., Hummel R., Wilken-Schmitz A., Tegeder I., Schäfer M.K.E. (2024). Selective neuronal expression of progranulin is sufficient to provide neuroprotective and anti-inflammatory effects after traumatic brain injury. J Neuroinflammation.

[43] Villapol S., Yaszemski A.K., Logan T.T., Sánchez-Lemus E., Saavedra J.M., Symes A.J. (2012). Candesartan, an angiotensin II AT1-Receptor blocker and PPAR-γ agonist, reduces lesion volume and improves motor and memory function after traumatic brain injury in mice. Neuropsychopharmacology.

[44] Subramanian A., Tamayo P., Mootha V.K., Mukherjee S., Ebert B.L., Gillette M.A. (2005). Gene set enrichment analysis: a knowledge-based approach for interpreting genome-wide expression profiles. Proc Natl Acad Sci U S A.

[45] Joo H., Bae J., Park J.-W., Lee B.-J., Lee B.D., Bu Y. (2021). Modified protocol to enable the study of hemorrhage and hematoma in a traumatic brain injury mouse model. Front Neurol.

[46] Kamali A., Dieckhaus L.A., Peters E.C., Preszler C.A., Witte R.S., Pires P.W. (2023). Ultrasound, photoacoustic, and magnetic resonance imaging to study hyperacute pathophysiology of traumatic and vascular brain injury. J Neuroimag.

[47] Badaut J., Adami A., Huang L., Obenaus A. (2020). Noninvasive magnetic resonance imaging stratifies injury severity in a rodent model of male juvenile traumatic brain injury. J Neurosci Res.

[48] Zusman B.E., Wu Y., Kochanek P.M., Vagni V.E., Janesko-Feldman K., Gerzanich V. (2023). Precision effects of glibenclamide on MRI endophenotypes in clinically relevant Murine Traumatic Brain injury. Crit Care Med.

[49] Le L.H.D., Eliseeva S., Plunk E., Kara-Pabani K., Li H., Yarovinsky F. (2025). The microglial response to inhibition of Colony-stimulating-factor-1 receptor by PLX3397 differs by sex in adult mice. Cell Rep.

[50] Easley-Neal C., Foreman O., Sharma N., Zarrin A.A., Weimer R.M. (2019). CSF1R ligands IL-34 and CSF1 are differentially required for Microglia development and maintenance in white and gray matter brain regions. Front Immunol.

[51] Johnson N.R., Yuan P., Castillo E., Lopez T.P., Yue W., Bond A. (2023). CSF1R inhibitors induce a sex-specific resilient microglial phenotype and functional rescue in a tauopathy mouse model. Nat Commun.

[52] Villapol S., Loane D.J., Burns M.P. (2017). Sexual dimorphism in the inflammatory response to traumatic brain injury. Glia.

[53] Gölz C., Kirchhoff F.P., Westerhorstmann J., Schmidt M., Hirnet T., Rune G.M. (2019). Sex hormones modulate pathogenic processes in experimental traumatic brain injury. J Neurochem.

[54] Clevenger A.C., Kim H., Salcedo E., Yonchek J.C., Rodgers K.M., Orfila J.E. (2018). Endogenous sex steroids dampen neuroinflammation and improve outcome of traumatic brain injury in mice. J Mol Neurosci.

[55] Doran S.J., Ritzel R.M., Glaser E.P., Henry R.J., Faden A.I., Loane D.J. (2019). Sex differences in acute neuroinflammation after experimental traumatic Brain injury are mediated by infiltrating Myeloid cells. J Neurotrauma.

[56] Liu X., Nemeth D.P., McKim D.B., Zhu L., DiSabato D.J., Berdysz O. (2019). Cell-Type-Specific interleukin 1 receptor 1 signaling in the brain regulates distinct neuroimmune activities. Immunity.

[57] Ching S., He L., Lai W., Quan N. (2005). IL-1 type I receptor plays a key role in mediating the recruitment of leukocytes into the central nervous system. Brain Behav Immun.

[58] Passaro A.P., Lebos A.L., Yao Y., Stice S.L. (2021). Immune response in neurological pathology: emerging role of central and peripheral immune crosstalk. Front Immunol.

[59] Shichita T., Sugiyama Y., Ooboshi H., Sugimori H., Nakagawa R., Takada I. (2009). Pivotal role of cerebral interleukin-17-producing gammadeltaT cells in the delayed phase of ischemic brain injury. Nat Med.

[60] Dong T., Zhi L., Bhayana B., Wu M.X. (2016). Cortisol-induced immune suppression by a blockade of lymphocyte egress in traumatic brain injury. J Neuroinflammation.

[61] Kleinschnitz C., Schwab N., Kraft P., Hagedorn I., Dreykluft A., Schwarz T. (2010). Early detrimental T-cell effects in experimental cerebral ischemia are neither related to adaptive immunity nor thrombus formation. Blood.

[62] Krämer T.J., Hack N., Brühl T.J., Menzel L., Hummel R., Griemert E.V. (2019). Depletion of regulatory T cells increases T cell brain infiltration, reactive astrogliosis, and interferon-γ gene expression in acute experimental traumatic brain injury. J Neuroinflammation.

[63] Gan Y., Liu Q., Wu W., Yin J.X., Bai X.F., Shen R. (2014). Ischemic neurons recruit natural killer cells that accelerate brain infarction. Proc Natl Acad Sci USA.

[64] Cho K.S., Lee E.J., Kim J.N., Choi J.W., Kim H.Y., Han S.H. (2015). Proteinase 3 induces neuronal cell death through microglial activation. Neurochem Res.

[65] Krasnow S.M., Knoll J.G., Verghese S.C., Levasseur P.R., Marks D.L. (2017). Amplification and propagation of interleukin-1β signaling by murine brain endothelial and glial cells. J Neuroinflammation.

[66] Vincent J.C., Garnett C.N., Watson J.B., Higgins E.K., Macheda T., Sanders L. (2023). IL-1R1 signaling in TBI: assessing chronic impacts and neuroinflammatory dynamics in a mouse model of mild closed-head injury. J Neuroinflammation.

[67] Claeys W., Verhaege D., Van Imschoot G., Van Wonterghem E., Van Acker L., Amelinck L. (2023). Limitations of PLX3397 as a microglial investigational tool: peripheral and off-target effects dictate the response to inflammation. Front Immunol.

[68] Lévesque S.A., Paré A., Mailhot B., Bellver-Landete V., Kébir H., Lécuyer M.-A. (2016). Myeloid cell transmigration across the CNS vasculature triggers IL-1β–driven neuroinflammation during autoimmune encephalomyelitis in mice. J Exp Med.

[69] Sjöström E.O., Culot M., Leickt L., Åstrand M., Nordling E., Gosselet F. (2021). Transport study of interleukin-1 inhibitors using a human *in vitro* model of the blood-brain barrier. Brain Behav Immun Health.

[70] Somebang K., Rudolph J., Imhof I., Li L., Niemi E.C., Shigenaga J. (2021). CCR2 deficiency alters activation of microglia subsets in traumatic brain injury. Cell Rep.

[71] Fritsch L.E., Kelly C., Leonard J., de Jager C., Wei X., Brindley S. (2024). STING-Dependent signaling in microglia or peripheral immune cells orchestrates the early inflammatory response and influences brain injury outcome. J Neurosci.

[72] Roselli F., Chandrasekar A., Morganti-Kossmann M.C. (2018). Interferons in traumatic brain and spinal cord injury: current evidence for translational application. Front Neurol.

[73] Todd B.P., Luo Z., Gilkes N., Chimenti M.S., Peterson Z., Mix M.R. (2023). Selective neuroimmune modulation by type I interferon drives neuropathology and neurologic dysfunction following traumatic brain injury. Acta Neuropatho Commun.

[74] Barrett J.P., Henry R.J., Shirey K.A., Doran S.J., Makarevich O.D., Ritzel R.M. (2020). Interferon-β plays a detrimental role in experimental traumatic brain injury by enhancing neuroinflammation that drives chronic neurodegeneration. J Neurosci.

[75] Todd B.P., Chimenti M.S., Luo Z., Ferguson P.J., Bassuk A.G., Newell E.A. (2021). Traumatic brain injury results in unique microglial and astrocyte transcriptomes enriched for type I interferon response. J Neuroinflammation.

[76] Witcher K.G., Bray C.E., Chunchai T., Zhao F., O’Neil S.M., Gordillo A.J. (2021). Traumatic brain injury causes chronic cortical inflammation and neuronal dysfunction mediated by Microglia. J Neurosci.

[77] Fritsch L.E., Ju J., Gudenschwager Basso E.K., Soliman E., Paul S., Chen J. (2022). Type I interferon response is mediated by NLRX1-cGAS-STING signaling in brain injury. Front Mol Neurosci.

[78] Abdullah A., Zhang M., Frugier T., Bedoui S., Taylor J.M., Crack P.J. (2018). STING-mediated type-I interferons contribute to the neuroinflammatory process and detrimental effects following traumatic brain injury. J Neuroinflammation.

[79] Karve I.P., Zhang M., Habgood M., Frugier T., Brody K.M., Sashindranath M. (2016). Ablation of Type-1 IFN signaling in hematopoietic cells confers protection following traumatic brain injury. eNeuro.

[80] Wangler L.M., Bray C.E., Packer J.M., Tapp Z.M., Davis A.C., O’Neil S.M. (2022). Amplified gliosis and interferon-associated inflammation in the aging brain following diffuse traumatic brain injury. J Neurosci.

[81] Petersen A., Soderstrom M., Saha B., Sharma P. (2021). Animal models of traumatic brain injury: a review of pathophysiology to biomarkers and treatments. Exp Brain Res.

[82] Xiong Y., Mahmood A., Chopp M. (2013). Animal models of traumatic brain injury. Nat Rev Neurosci.

[83] Newell E.A., Todd B.P., Luo Z., Evans L.P., Ferguson P.J., Bassuk A.G. (2020). A mouse model for juvenile, lateral fluid percussion brain injury reveals sex-dependent differences in neuroinflammation and functional recovery. J Neurotrauma.

[84] Jha R.M., Rajasundaram D., Sneiderman C., Schlegel B.T., O’Brien C., Xiong Z. (2024). A single-cell atlas deconstructs heterogeneity across multiple models in murine traumatic brain injury and identifies novel cell-specific targets. Neuron.

[85] Macheda T., Andres M.R., Sanders L., Roberts K.N., Shahidehpour R.K., Morganti J.M. (2024). Old age exacerbates white matter neuroinflammation and cognitive deficits following closed-head injury, particularly in female mice. Neurotrauma Rep.

[86] Walker K.R., Tesco G. (2013). Molecular mechanisms of cognitive dysfunction following traumatic brain injury. Front Aging Neurosci.

[87] Lennon M.J., Brooker H., Creese B., Thayanandan T., Rigney G., Aarsland D. (2023). Lifetime traumatic brain injury and cognitive domain deficits in late life: the PROTECT-TBI cohort study. J Neurotrauma.

[88] Campos-Pires R., Hirnet T., Valeo F., Ong B.E., Radyushkin K., Aldhoun J. (2019). Xenon improves long-term cognitive function, reduces neuronal loss and chronic neuroinflammation, and improves survival after traumatic brain injury in mice. Br J Anaesth.

[89] Pöttker B., Stöber F., Hummel R., Angenstein F., Radyushkin K., Goldschmidt J. (2017). Traumatic brain injury causes long-term behavioral changes related to region-specific increases of cerebral blood flow. Brain Struct Funct.

[90] Griffiths D.R., Law L.M., Young C., Fuentes A., Truran S., Karamanova N. (2022). Chronic cognitive and cerebrovascular function after mild traumatic brain injury in rats. J Neurotrauma.

[91] Attilio P.J., Snapper D.M., Rusnak M., Isaac A., Soltis A.R., Wilkerson M.D. (2021). Transcriptomic analysis of mouse brain after traumatic brain injury reveals that the angiotensin receptor blocker candesartan acts through novel pathways. Front Neurosci.

[92] Lipponen A., Paananen J., Puhakka N., Pitkänen A. (2016). Analysis of post-traumatic brain injury gene expression signature reveals tubulins, Nfe2l2, Nfkb, Cd44, and S100a4 as treatment targets. Sci Rep.

[93] Lipponen A., El-Osta A., Kaspi A., Ziemann M., Khurana I., Kn H. (2018). Transcription factors Tp73, Cebpd, Pax6, and Spi1 rather than DNA methylation regulate chronic transcriptomics changes after experimental traumatic brain injury. Acta Neuropathol Commun.

[94] Arneson D., Zhang G., Ahn I.S., Ying Z., Diamante G., Cely I. (2022). Systems spatiotemporal dynamics of traumatic brain injury at single-cell resolution reveals humanin as a therapeutic target. Cell Mol Life Sci.

[95] Ciechanowska A., Ciapała K., Pawlik K., Oggioni M., Mercurio D., De Simoni M.G. (2020). Initiators of classical and Lectin complement pathways are differently engaged after traumatic brain injury-time-dependent changes in the cortex, striatum, thalamus and hippocampus in a mouse model. Int J Mol Sci.

[96] Gottlieb A., Toledano-Furman N., Prabhakara K.S., Kumar A., Caplan H.W., Bedi S. (2022). Time dependent analysis of rat microglial surface markers in traumatic brain injury reveals dynamics of distinct cell subpopulations. Sci Rep.

[97] Makinde H.M., Just T.B., Gadhvi G.T., Winter D.R., Schwulst S.J. (2020). Microglia adopt longitudinal transcriptional changes after traumatic brain injury. J Surg Res.

[98] Kumar A., Alvarez-Croda D.M., Stoica B.A., Faden A.I., Loane D.J. (2016). Microglial/macrophage polarization dynamics following traumatic brain injury. J Neurotrauma.

[99] Puntambekar S.S., Saber M., Lamb B.T., Kokiko-Cochran O.N. (2018). Cellular players that shape evolving pathology and neurodegeneration following traumatic brain injury. Brain Behav Immun.

[100] Ghiam M.K., Patel S.D., Hoffer A., Selman W.R., Hoffer B.J., Hoffer M.E. (2021). Drug repurposing in the treatment of traumatic brain injury. Front Neurosci.

[101] Margulies S., Anderson G., Atif F., Badaut J., Clark R., Empey P. (2016). Combination therapies for traumatic brain injury: retrospective considerations. J Neurotrauma.

[102] Somayaji M.R., Przekwas A.J., Gupta R.K. (2018). Combination therapy for multi-target manipulation of secondary brain injury mechanisms. Curr Neuropharmacol.

