# Supplementary material for: CSF1R and IL1R1 inhibitors synergistically attenuate the early pathogenesis of traumatic brain injury in mice
Source: Neurotherapeutics. 2025 Nov 7;23(1):e00787. doi: 10.1016/j.neurot.2025.e00787 (PMC12976530; doi:10.1016/j.neurot.2025.e00787)
Supplement: Multimedia component 1 [file mmc1.docx]

**Fig. S1: Food consumption**


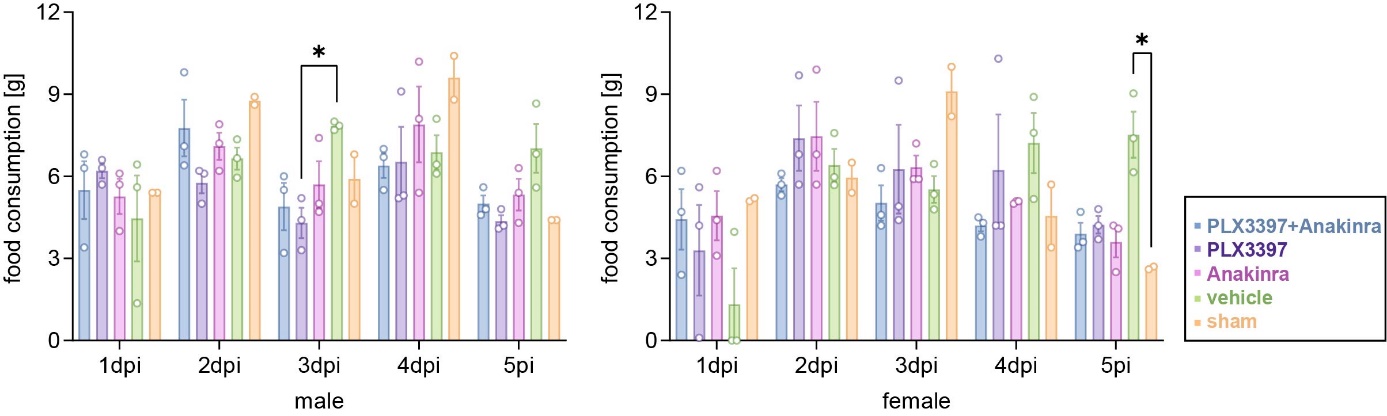


Fig. S1: Food consumption in male and female mice from 5d survival after TBI/sham. Data points represent individual cages (two animals per cage), TBI: n=3 cages per group, sham: n=2 cages per group, and data are expressed as mean ± SEM. Two-Way ANOVA followed by Holm-Šídák multiple comparisons test, **p* < 0.05.

**Fig. S2: Body weights**


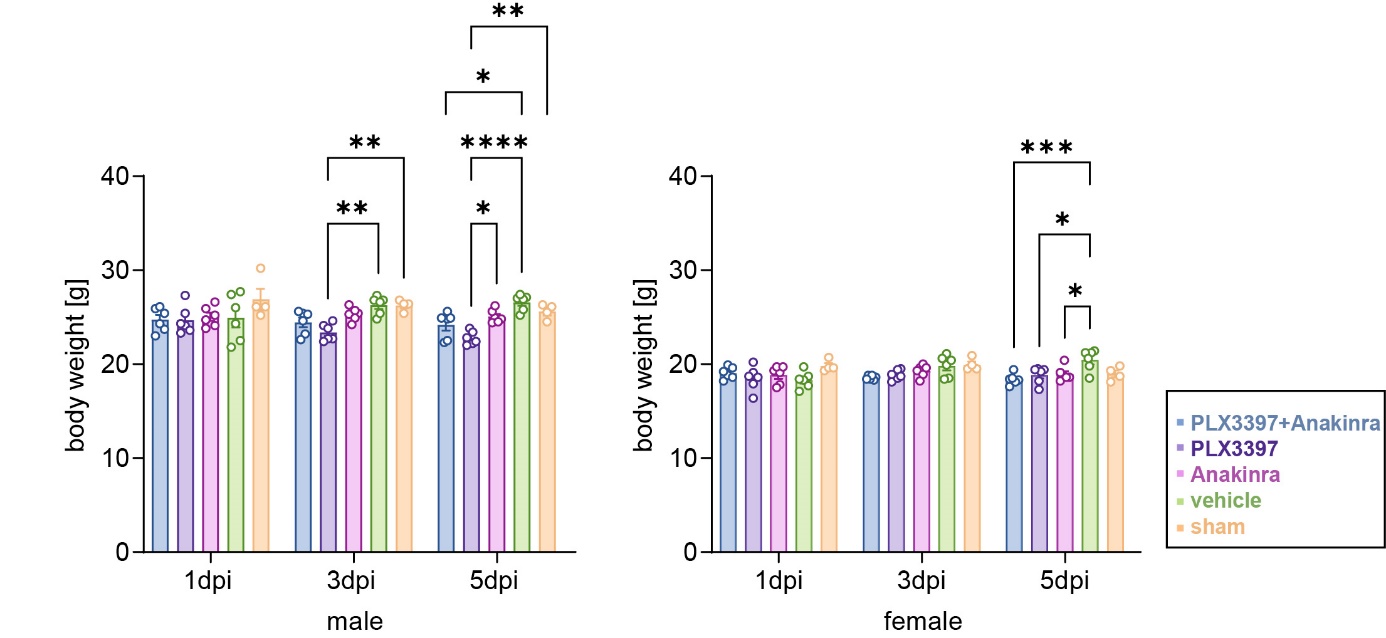


Fig. S2: Body weight of male and female mice over 5 days after TBI/sham. Male mice treated with PLX3397 plus Anakinra exhibited a reduced body weight compared to vehicle at 5 dpi. PLX3397 monotherapy showed reduced body weight at 3 dpi compared to vehicle or sham, and at 5 dpi compared to Anakinra, vehicle, and sham. Female mice treated with vehicle exhibited an increased body weight compared to other TBI treatment groups at 5 dpi. Data points represent individual mice, TBI: n=6 per group, sham: n=4 per group, and data are expressed as mean ± SEM. Two-Way ANOVA followed by Holm-Šídák multiple comparisons test, **p* < 0.05, ***p* < 0.01, ****p* < 0.001, *****p* < 0.0001.

**Fig. S3: Principal component analysis of male mice**


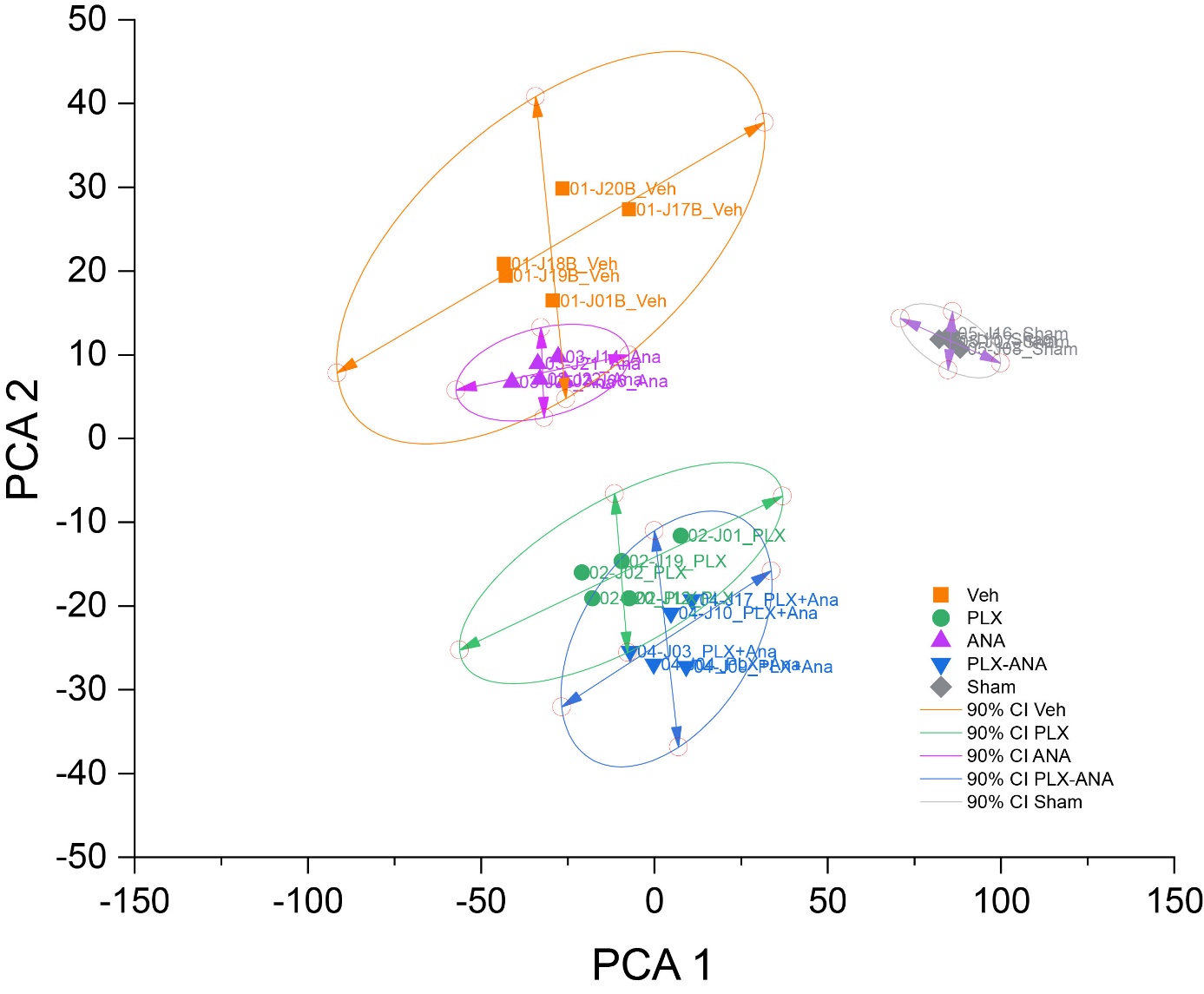


Fig. S3: Principal component analysis (PCA) of male mice RNA-sequencing data to visualize variation between individual mice and experimental groups. Data points represent individual mice (n=4-5 per group), experimental groups are circled to visualize clustering.

**Fig. S4: Heatmap showing significantly regulated genes of experimental groups**


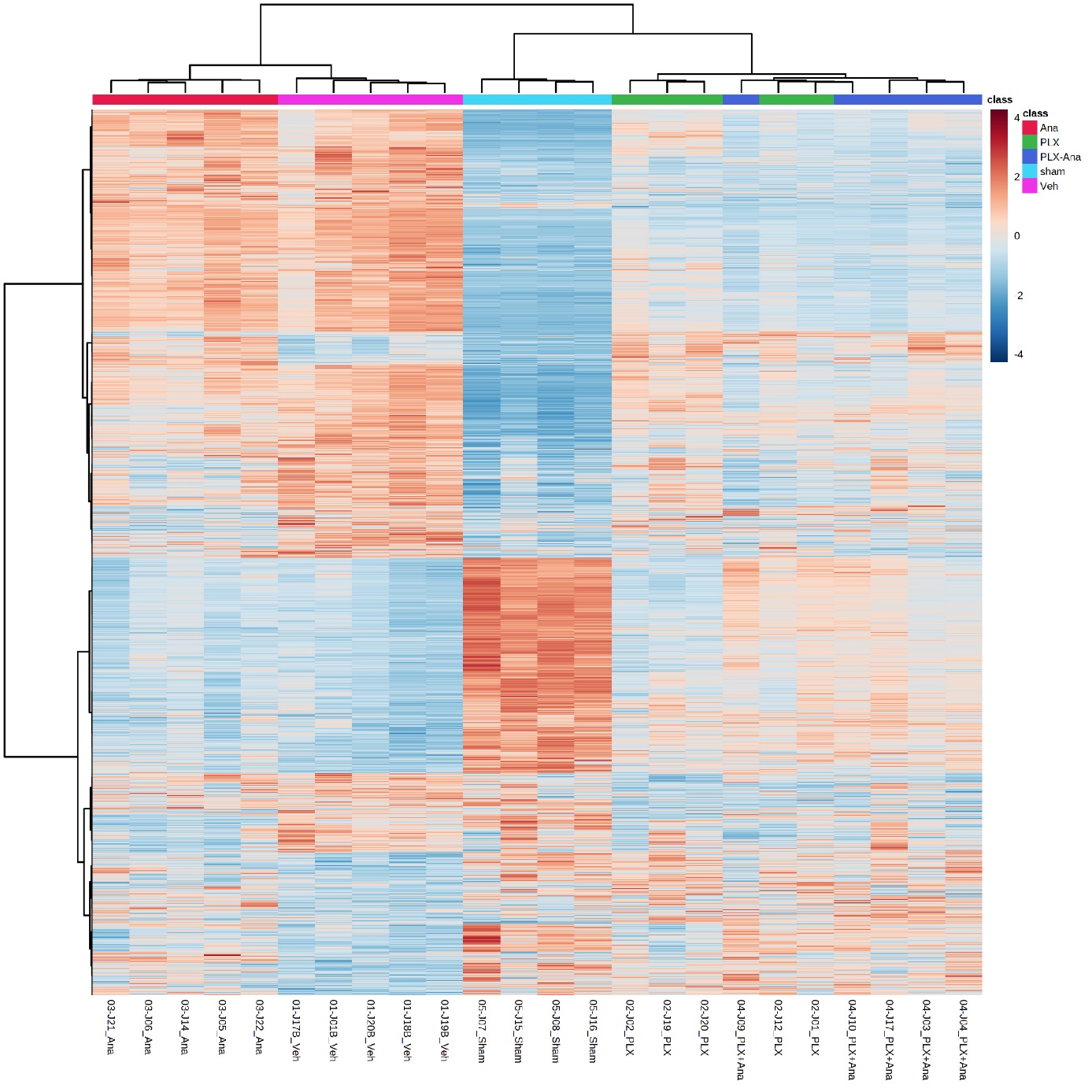


Fig. S4: Heatmap showing significantly regulated genes based on ANOVA of male experimental groups (n=4-5 per group). Hierarchical clustering of genes is shown (Eulidean-distance metrics, Ward-method) red to blue color indicates high to low relative expression.

**Fig. S5: Heatmap showing Top 100 significantly regulated genes in TBI groups**

**
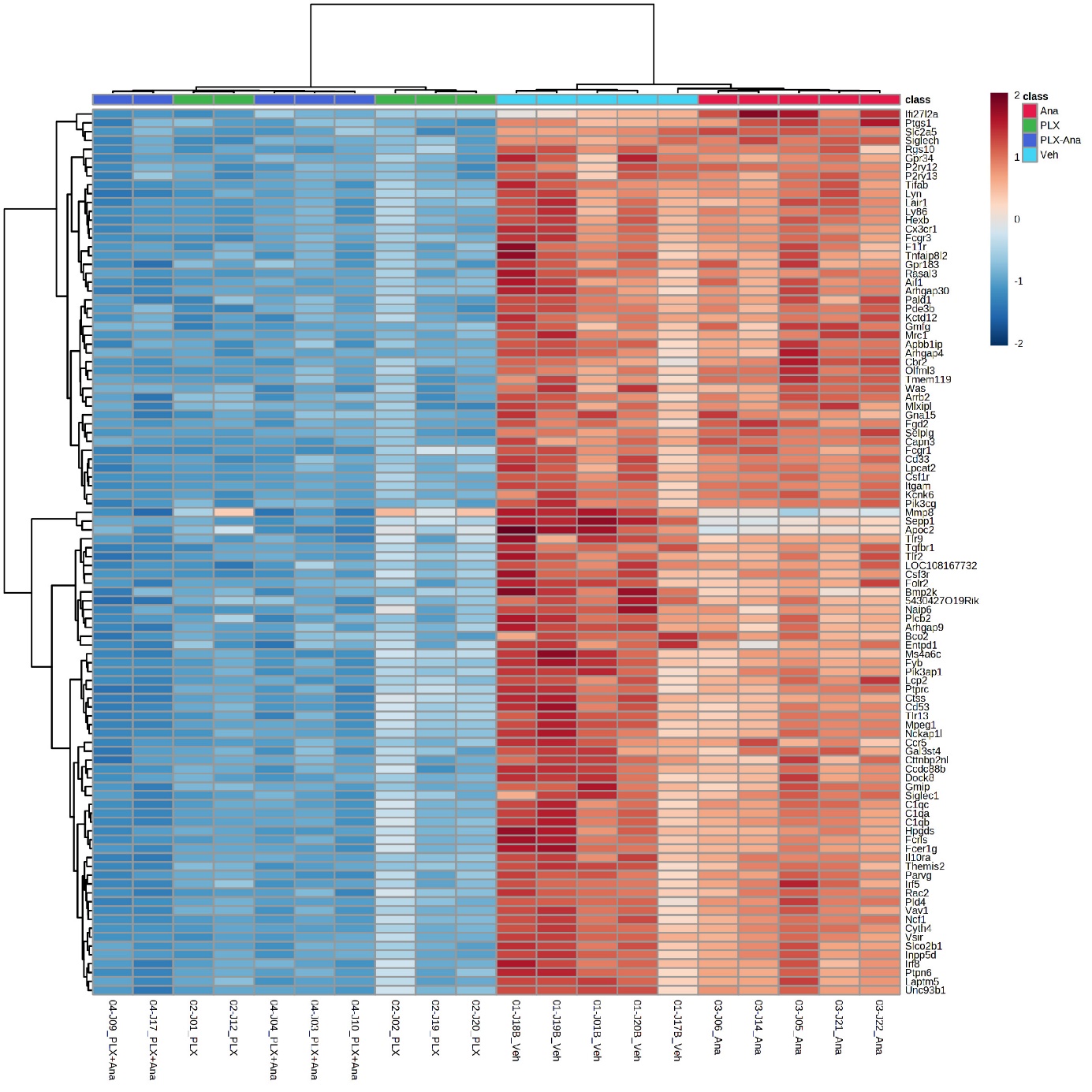
**

Fig. S5: Heatmap showing Top 100 significantly regulated genes based on ANOVA of male TBI groups of male mice (n=5 per group). Hierarchical clustering of genes is shown (Eulidean-distance metrics, Ward-method) red to blue color indicates high to low relative expression.

**Fig. S6: Volcano plots showing DEGs in TBI groups versus sham**


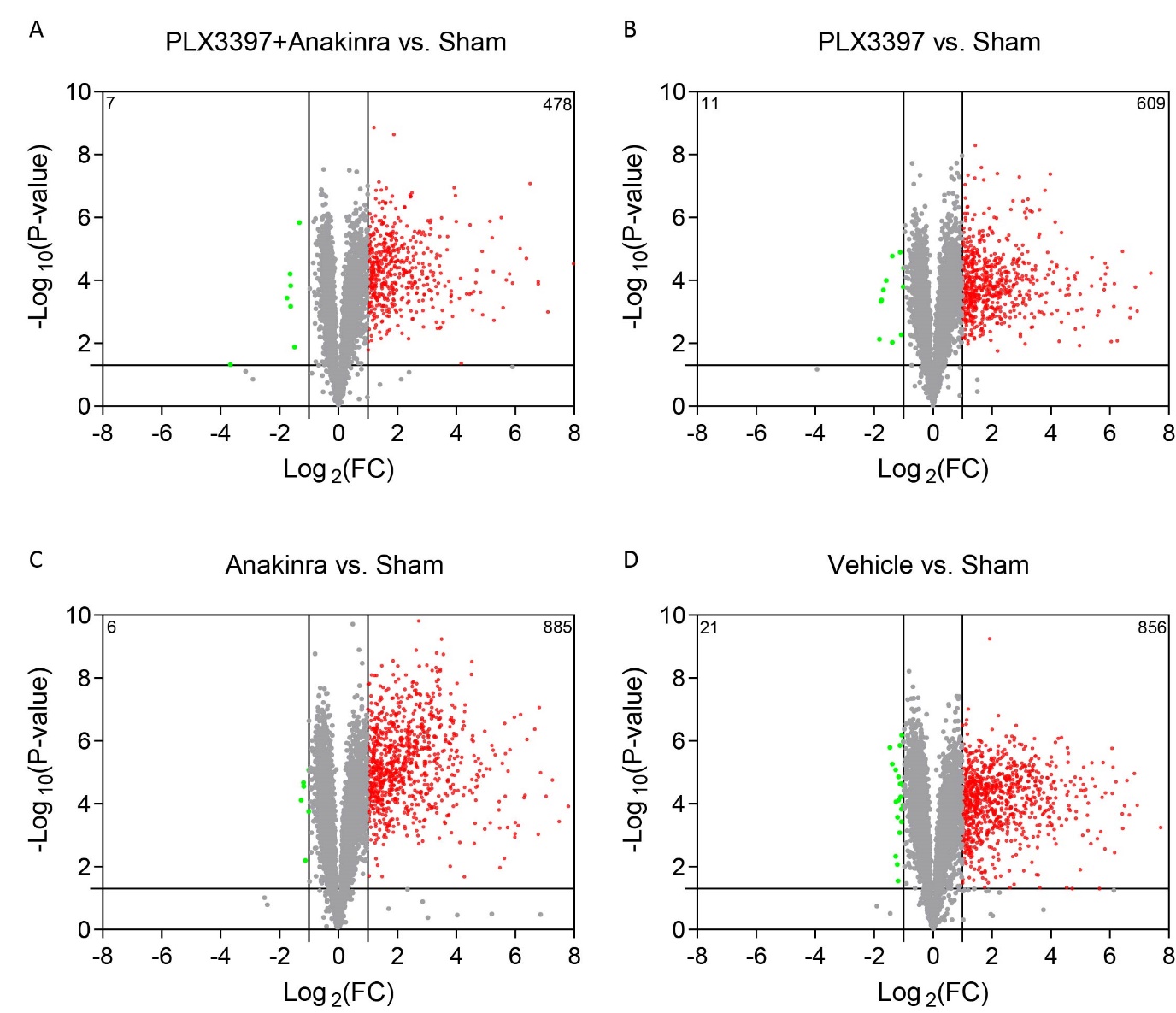


Fig. S6: (A-D) Volcano plots showing DEGs in TBI groups versus sham (n=5 per group) with the criterion: absolute difference > 4, fold change > 2, p < 0.05. The X-axis represents the log2-transformed fold change (log2 FC) and the Y-axis represents the negative log10-transformed *p*-value. In all four comparisons, significantly up- or down-regulated DEGs (red dots = upregulated, green dots = downregulated) and non-significantly regulated genes (grey dots) are indicated.

**Fig. S7: Enrichment plots of immune and inflammation-associated processes**


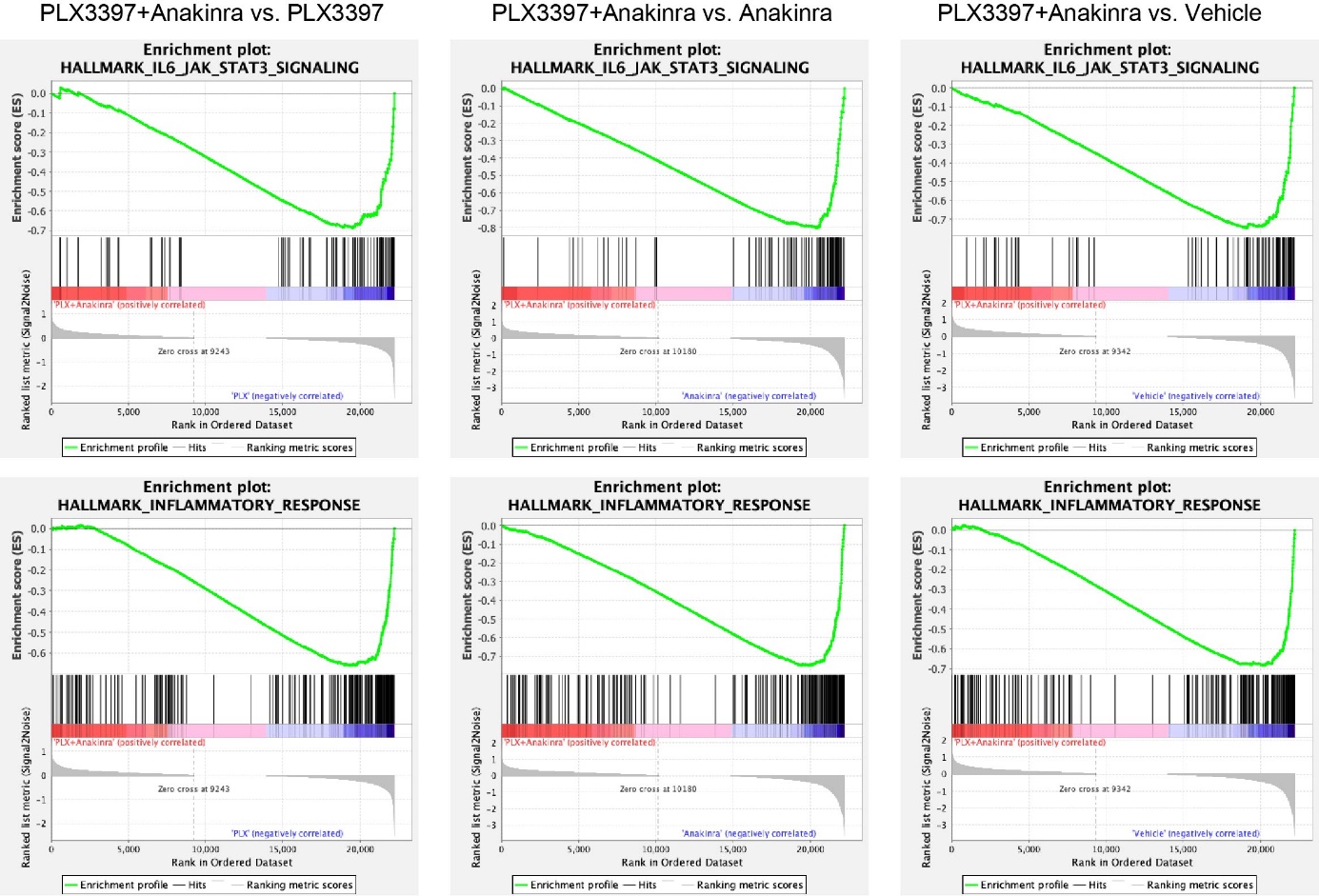


Fig. S7: Enrichment plots, generated using GSEA 4.1.0, show the immune and inflammation-associated hallmark gene sets in combination of PLX3397 plus Anakinra versus mice treated with PLX3397, Anakinra monotherapy or vehicle (n=5 per group). The green line chart represents the gene ES and the peak represents maximum enrichment. The vertical black bars represent individual genes of the gene set.

**Fig. S8: Enrichment plots of hallmark-IFNγ response-related gene sets**


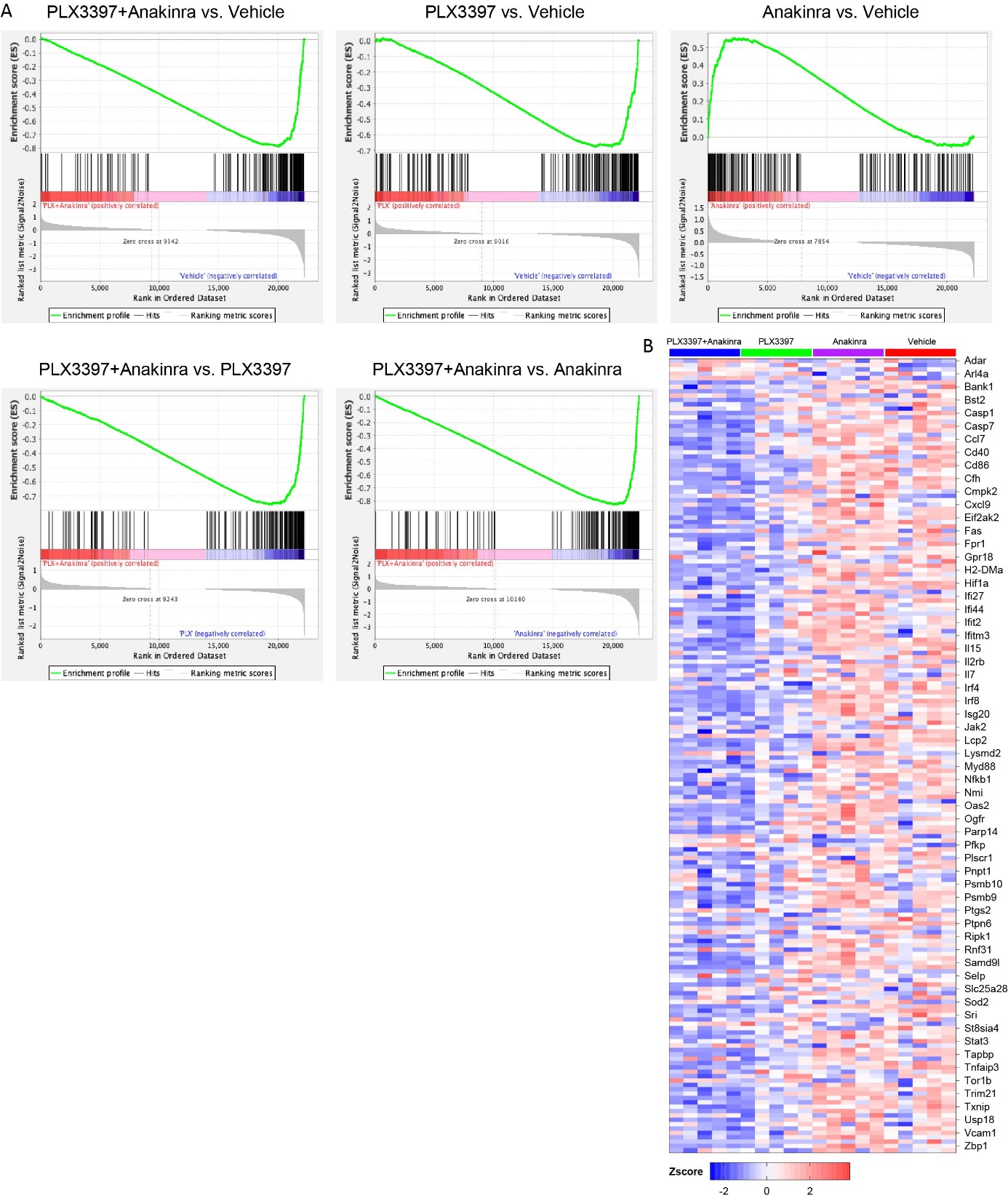


Fig. S8: (A) Enrichment plots, generated using GSEA 4.1.0, show the hallmark-IFNγ response-related gene sets in TBI groups versus vehicle (n=5 per group). The green line chart represents the gene ES and the peak represents maximum enrichment. The vertical black bars represent individual genes of the gene set. (B) Heatmap showing relative expression levels (Zscores) of 182 genes belonging to hallmark “IFNγ response”, red to blue color indicates high to low expression.

**Fig. S9: Enrichment plots of neurotransmission- or synapse-related GOBPs**


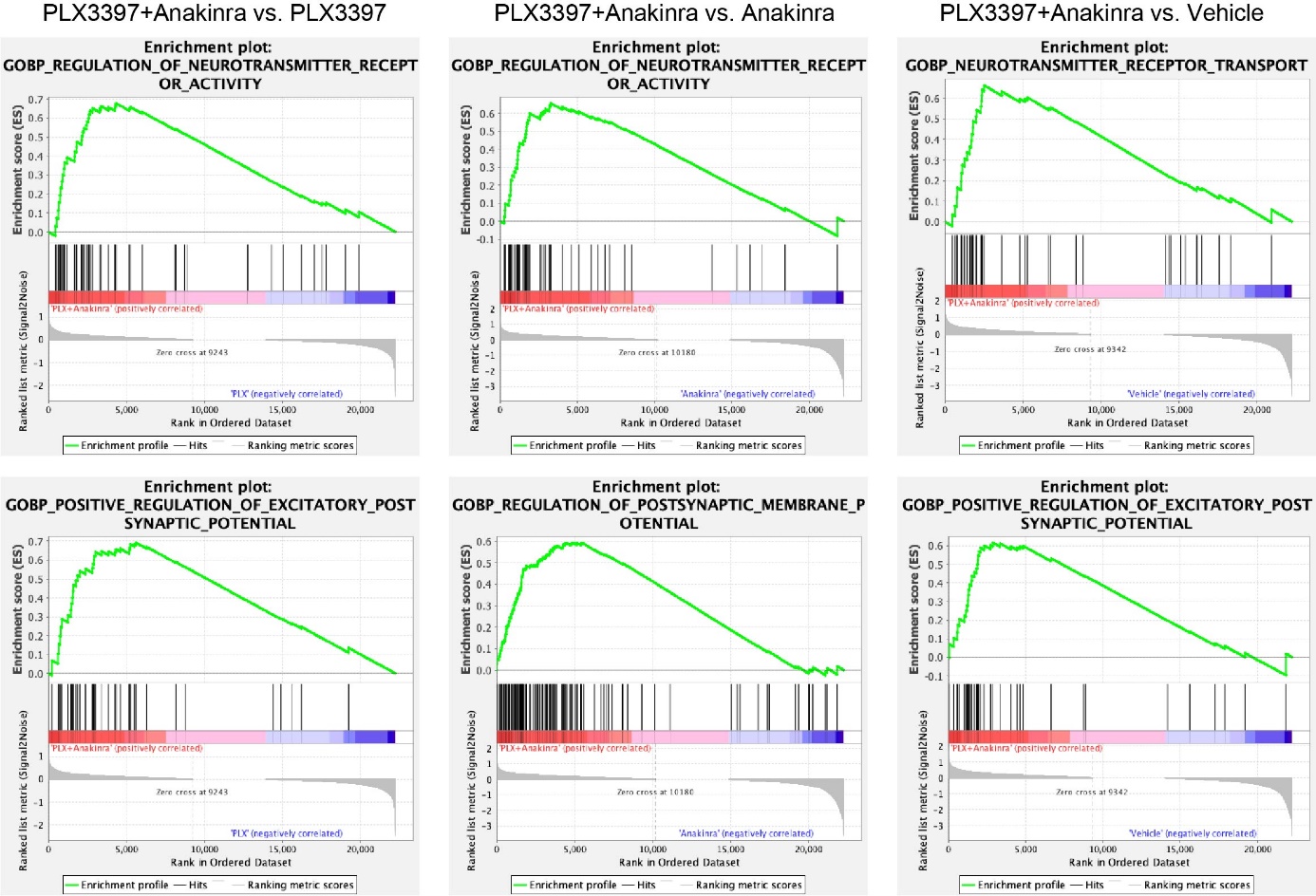


Fig. S9: Enrichment plots, generated using GSEA 4.1.0, show the neurotransmission- or synapse-related GOBPs in combination of PLX3397 plus Anakinra versus mice treated with vehicle, PLX3397 or Anakinra monotherapy (n=5 per group). The green line chart represents the gene ES and the peak represents maximum enrichment. The vertical black bars represent individual genes of the gene set.

**Fig. S10: Immunofluorescence staining of CD68^+^, GFAP^+^, and CD45^+^ cells in CCI vs. sham mice**


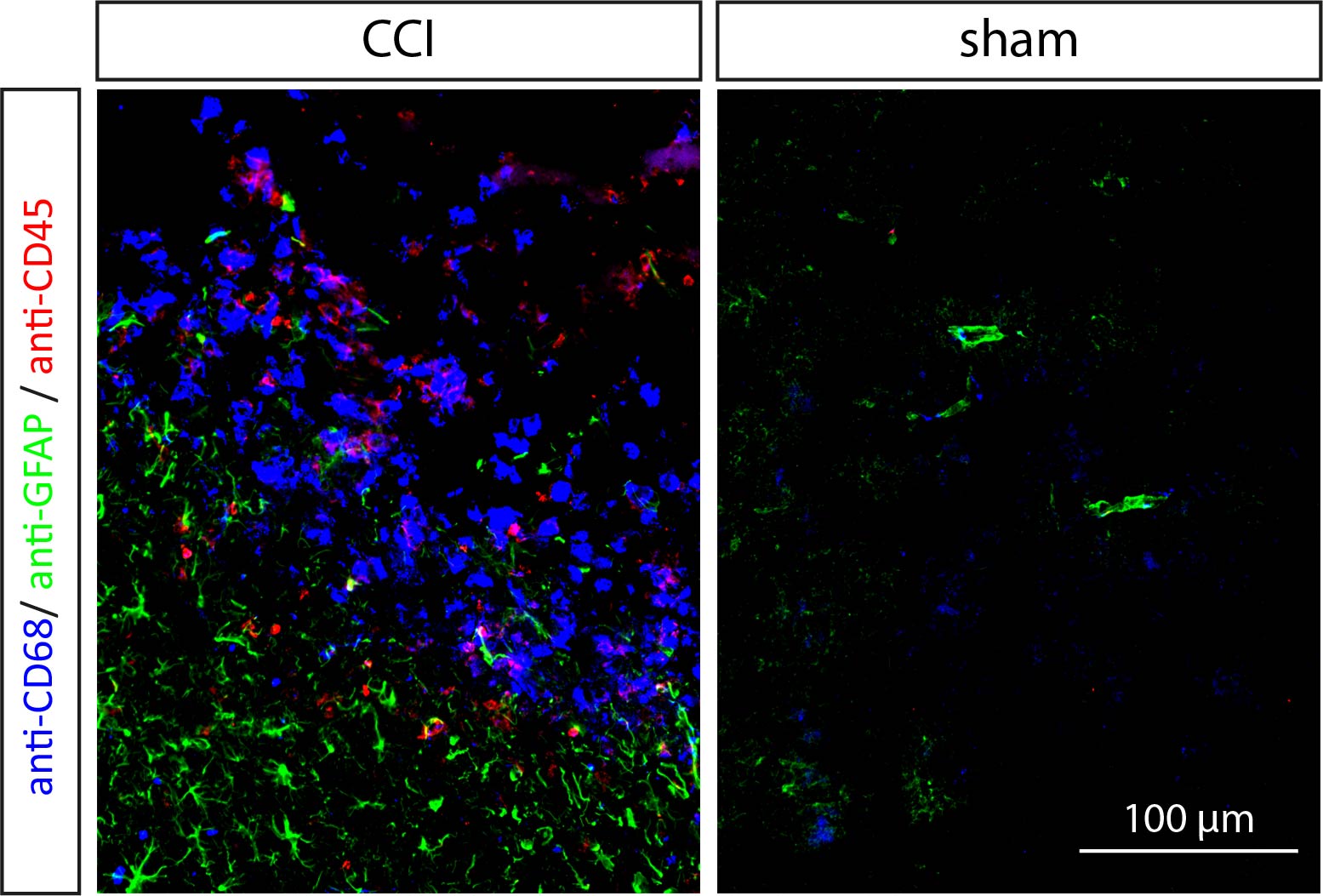


Fig. S10: Triple immunostaining in CCI vs. sham mice of the ipsilesional cortex at 5 dpi using antibodies specific to CD68, GFAP, or CD45 to visualize microglia, astrocytes and leukocytes, respectively (scale: 100 µm, Bregma -0.86 mm).

**Table S1: Antibodies**

| Primary antibodies | | | | | |
| --- | --- | --- | --- | --- | --- |
| Primary antibody | Host | | IHC, dilution | Manufacturer | RRID |
| CD68 BV421 | Rat | | 1:250 | BD Horizon | AB_2744447 |
| GFAP | Rabbit | | 1:1000 | Agilent Dako | AB_10013382 |
| CD45 | Rat | | 1: 500 | eBioscience | AB_467252 |
| Osteopontin | Rabbit | | 1:250 | Proteintech | AB_2783651 |
| Secondary antibodies | | | | | |
| Fluorophore | Host | Species | IHC, dilution | Manufacturer | RRID |
| Alexa 568 | Goat | Rat | 1:500 | Invitrogen/Life Technologies | AB_2534121 |
| Alexa 568 | Donkey | Rabbit | 1:500 |  | AB_2534017 |
| Alexa 633 | Goat | Rabbit | 1:500 |  | AB_2535731 |

**Table S2: Gene list of Venn diagram with related STRING-DB permalink**

PLX3397+Anakinra / PLX3397 / Anakinra vs. vehicle (criterion: absolute difference > 4, fold change > 2, *p*-value < 0.05)

**S2.1: PLX3397+Anakinra / PLX3397 / Anakinra: 9 genes**

| Postn | Mfap4 | Lum | H19 | Col5a2 | Col12a1 | Aspn | Crabp1 | Arg1 |  |
| --- | --- | --- | --- | --- | --- | --- | --- | --- | --- |

STRING-DB: <https://version-12-0.string-db.org/cgi/network?networkId=bmBXczhb5QjF>

**
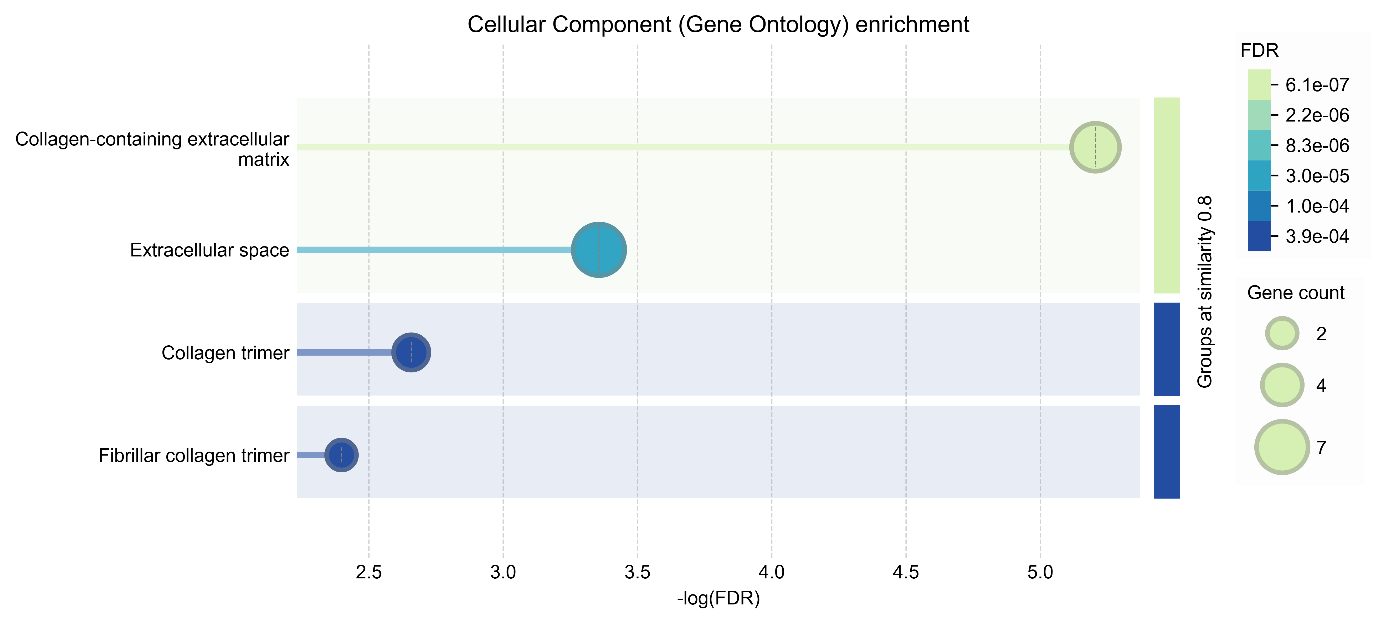
**

**S2.2: PLX3397+Anakinra / PLX3397: 158 genes**

| P2ry6 | Ctss | Ccl6 | Cd74 | Siglech | Tgfb1 | Fcgr3 | Ms4a6c | Bcl2a1b | Alox5ap |
| --- | --- | --- | --- | --- | --- | --- | --- | --- | --- |
| Gal3st4 | AB124611 | Lpxn | Hcls1 | C1qb | Cybb | Fcrls | Lat2 | H2-Ab1 | Kcnk6 |
| Ifi30 | Selplg | Irf5 | Ang | Apoc2 | Snx20 | Ccl9 | Cbr2 | Gna15 | Arl11 |
| Ly86 | Laptm5 | Havcr2 | Ch25h | Gpr34 | Myo1f | H2-Eb1 | Ikzf1 | Samsn1 | Hbb-bs |
| Tmem119 | Ms4a6d | Pik3ap1 | C1qa | Arhgap30 | Ncf4 | Tlr2 | Tlr13 | Hexb | Susd3 |
| Stab1 | H2-Aa | Dock8 | Itgbl1 | C5ar1 | Bin2 | Vav1 | Arhgap9 | Rab32 | Dock2 |
| Plcg2 | Unc93b1 | Cyth4 | Rac2 | Hba-a1 | P2ry13 | Hmha1 | Lrrc25 | Inpp5d | Hbb-bt |
| Itgb2 | Tifab | Nfam1 | Grn | Arhgap4 | Apoc1 | Gpr65 | Pld4 | Fcgr2b | Mgp |
| Cx3cr1 | Gpr84 | Olfml3 | Cd52 | Parvg | Gpsm3 | Sash3 | Syk | Capn3 | Alas2 |
| Folr2 | Ptpn6 | Cmtm7 | Aif1 | Cd33 | Tlr7 | Hk2 | Fes | Hba-a2 | Fcer1g |
| Crybb1 | Lcp2 | Ltc4s | Btk | Cd86 | Ptpn18 | Csf3r | Lair1 | Hpgds | Fgd2 |
| Naip5 | Fcgr1 | Abcc3 | Ccr5 | P2ry12 | Pf4 | Pycard | Ncf2 | Il10ra | Ms4a6b |
| Ucp2 | Nckap1l | Ifi27l2a | Msr1 | Ms4a4a | Apbb1ip | Tbxas1 | Slco2b1 | Tnfaip8l2 | Adgre1 |
| Fyb | Irf8 | Vsir | Mpeg1 | Gcnt1 | Ugt1a7c | Ms4a7 | Hk3 | Wfdc17 | F13a1 |
| Fermt3 | Lcp1 | Was | Mrc1 | Itgam | Lyl1 | Lyz2 | Clec7a | Rasal3 | Cd37 |
| Csf1r | Il21r | Siglec1 | C1qc | Hck | Cd84 | Ncf1 | Spi1 |  |  |

STRING-DB: <https://version-12-0.string-db.org/cgi/network?networkId=bz6SnhrMmCuu>


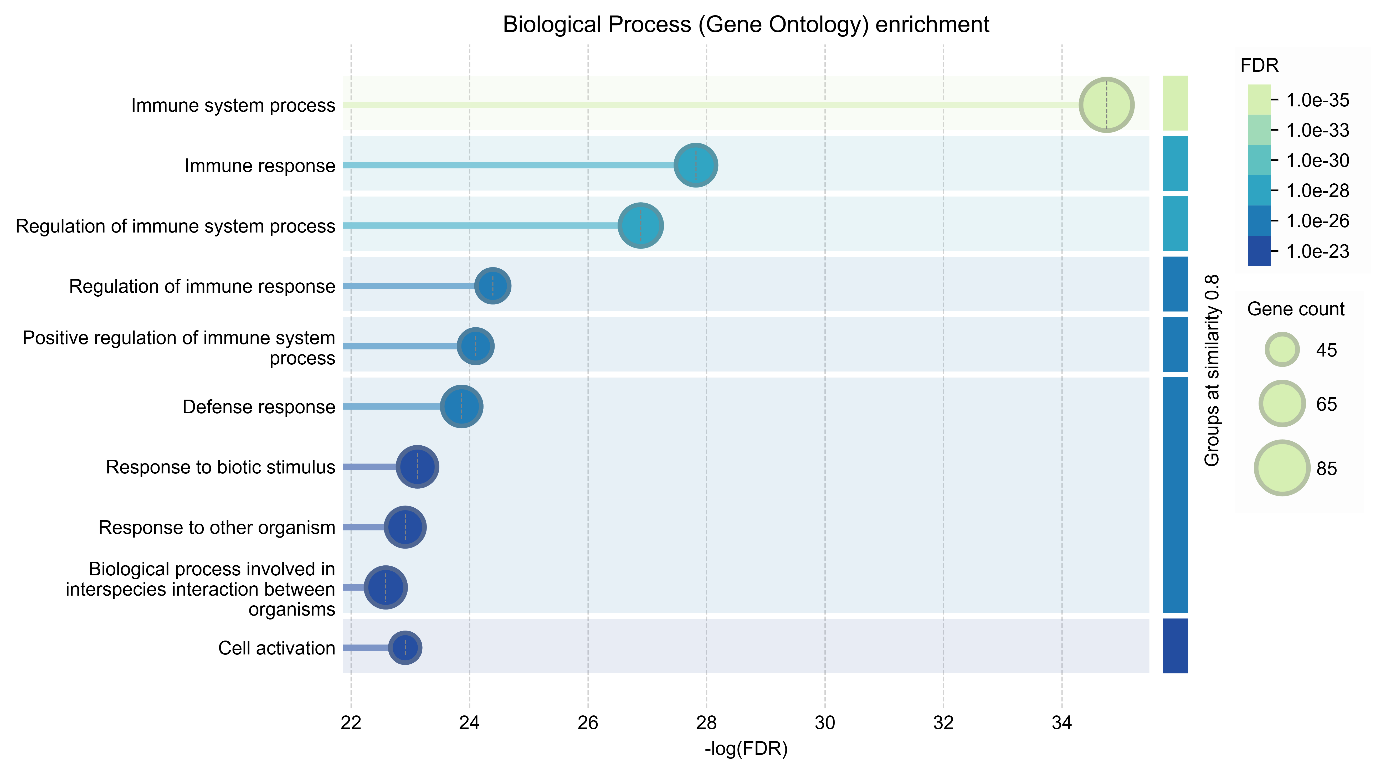


**S2.3: PLX3397+Anakinra / Anakinra: 6 genes**

| Eln | Lcn2 | Col3a1 | Col1a2 | Lrrc17 | Col1a1 |  |  |  |  |
| --- | --- | --- | --- | --- | --- | --- | --- | --- | --- |

STRING-DB: <https://version-12-0.string-db.org/cgi/network?networkId=bVx6iJ9L9ZIR>


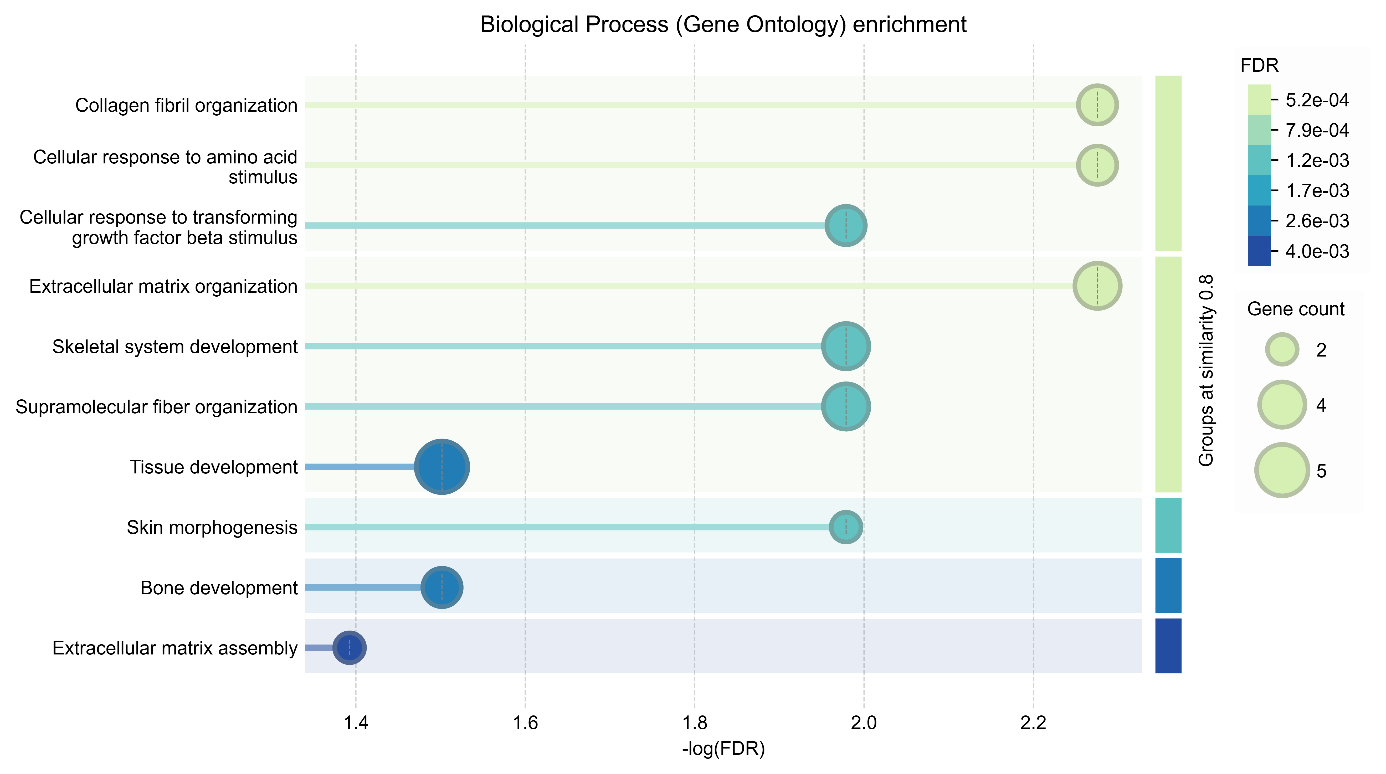


**S2.4: PLX3397+ Anakinra: 108 genes**

| Adap2 | Npl | Slfn8 | Igsf6 | Gmip | Tmem106a | Tyrobp | Slc11a1 | Slc15a3 | Slamf9 |
| --- | --- | --- | --- | --- | --- | --- | --- | --- | --- |
| Plin2_2 | Oas1a | Mafb | Casp1 | Clec4n | Rnase4 | Cxcr4 | Cyba | Cd48 | Gal |
| Fli1 | Lilrb4a | Pbk | Hvcn1 | Ly9 | Clec4d | Cd53 | Pik3cg | Sp110 | Phf11b |
| Cd300a | Ccl7 | Cd5l | Cebpa | Il4ra | Ccl12 | Spp1 | Il10rb | Ctsh | Pirb |
| Klhl6 | Ccdc88b | Lst1 | Tnfaip2 | Gusb | Clec5a | Neurl3 | Apobec1 | Ctsd | Hhex |
| C3 | AI607873 | Lyn | Cmklr1 | Slc7a7 | Fabp4 | Ccl8 | H2-DMb1 | Themis2 | Ptafr |
| Abca9 | Cfp | Tnfsf13 | Trim30a | Gpr157 | Cyp4f18 | Slc37a2 | Dnase2a | Hpse | Plek |
| Lilr4b | Cd300lf | Ifi204 | Cd68 | Csf2rb2 | Ctsc | Cd36 | Pik3r5 | Il2rg | Gpnmb |
| Adam8 | Lgals3 | Csf2rb | Cryba4 | Fcgr4 | Epsti1 | Srgn | Lpcat2 | Ctla2b | Tnfrsf1b |
| Lgmn | Fam46c | Hcst | Gngt2 | Cd180 | Cd72 | Ptprc | Cd300c2 | Adcy7 | Ccl3 |
| Acp5 | Rab7b | C3ar1 | Arhgap19 | Tmem37 | Slfn2 | Naip2 | Trem2 |  |  |

STRING-DB: <https://version-12-0.string-db.org/cgi/network?networkId=b6gXq9nQdEfD>


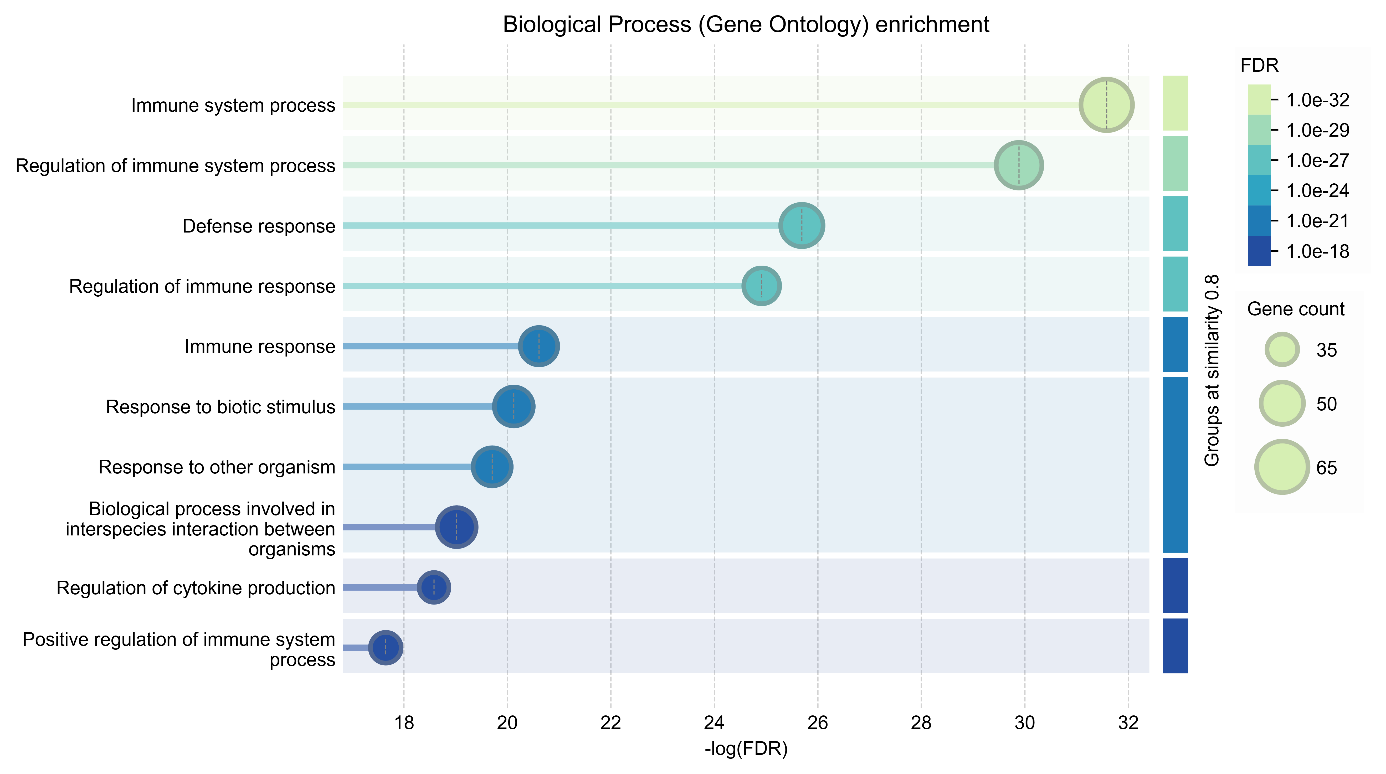


**S2.5: Anakinra: 12 genes**

| Col10a1 | Col6a3 | Thbs2 | Ctsk | Lrrc15 | Actg2 | Mmp12 | Ppic | Lox | Fbn1 |
| --- | --- | --- | --- | --- | --- | --- | --- | --- | --- |
| Rmrp | Loxl2 |  |  |  |  |  |  |  |  |

STRING-DB: <https://version-12-0.string-db.org/cgi/network?networkId=bupDKdqwwpRw>


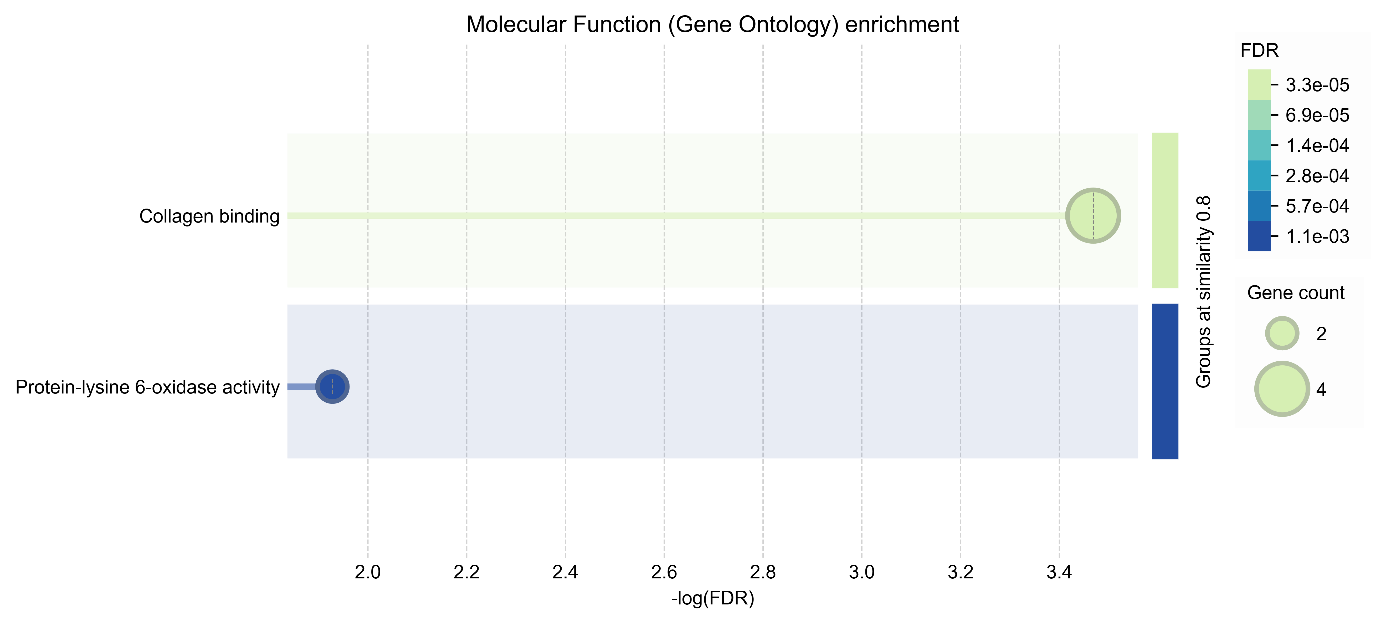


**Table S3:** **DEGs were analysed by STRING-pathway analyses**

| Comparison | STRING-DB permalink | Associated pathway |
| --- | --- | --- |
| PLX3397+Anakinra vs. vehicle | <https://version-12-0.string-db.org/cgi/network?networkId=bPzgifMDmHNr> | immune system process, immune response |
| PLX3397 vs. vehicle | <https://version-12-0.string-db.org/cgi/network?networkId=bsQ3zK84HGJ9> | immune system process, immune response |
| Anakinra vs. vehicle | <https://version-12-0.string-db.org/cgi/network?networkId=bmVVEoHo3MHc> | ECM, collagen synthesis |
| PLX3397+Anakinra vs. PLX3397 | <https://version-12-0.string-db.org/cgi/network?networkId=bXPK4HVjJpSy> | regulation of multicellular organismal processes, regulation of lipid transport |
| PLX3397+Anakinra vs. Anakinra | https://version-12-0.string-db.org/cgi/network?networkId=buSqQalhBlK4 | immune system process, immune response |

**Table S4. Statistics for Fig. 2**

two-way ANOVA (or mixed-effects models following outlier removal)

| Fig. 2A | | | | |
| --- | --- | --- | --- | --- |
| time*treatment: F (12, 69) = 11.61, p<0.0001  time: F (3, 69) = 205.8, p<0.0001  treatment: F (4, 23) = 30.41, p<0.0001 | | | | |
| PLX3397+Anakinra+TBI vs. PLX3397+TBI  pre-score: p>0.9999  1dpi: p=0.0113  3dpi: p=0.0021  5dpi: p=0.0043 | PLX3397+Anakinra+TBI vs. Anakinra+TBI  pre-score: p>0.9999  1dpi: p=0.5946  3dpi: p=0.1447  5dpi: p=0.0654 | PLX3397+Anakinra+TBI vs. vehicle+TBI  pre-score: p>0.9999  1dpi: p=0.0151  3dpi: p=0.0003  5dpi: p=0.0043 | PLX3397+TBI  vs. vehicle+TBI  pre-score: p>0.9999  1dpi: p=0.8793  3dpi: p=0.5440  5dpi: p>0.9999 | Anakinra+TBI  vs. vehicle+TBI  pre-score: p>0.9999  1dpi: p=0.1033  3dpi: p=0.0951  5dpi: p=0.6410 |
| Fig. 2B | | | | |
| time*treatment: F (12, 68) = 10.68, p<0.0001  time: F (3, 68) = 151.3, p<0.0001  treatment: F (4, 23) = 27.44, p<0.0001 | | | | |
| PLX3397+Anakinra+TBI vs. PLX3397+TBI  pre-score: p=0.9997  1dpi: p=0.0331  3dpi: p=0.2102  5dpi: p=0.0249 | PLX3397+Anakinra+TBI vs. Anakinra+TBI  pre-score: p=0.9998  1dpi: p=0.4559  3dpi: p=0.2102  5dpi: p=0.4559 | PLX3397+Anakinra+TBI vs. vehicle+TBI  pre-score: p=0.9997  1dpi: p=0.0007  3dpi: p<0.0001  5dpi: p<0.0001 | PLX3397+TBI  vs. vehicle+TBI  pre-score: p>0.9999  1dpi: p=0.3294  3dpi: p=0.0056  5dpi: p=0.1063 | Anakinra+TBI  vs. vehicle+TBI  pre-score: p=0.9997  1dpi: p=0.0070  3dpi: p=0.0043  5dpi: p<0.0001 |
| Fig. 2C | | | | |
| time*treatment: F (12, 69) = 1.245, p=0.2718  time: F (3, 69) = 2.473, p=0.0689  treatment: F (4, 23) = 0.8790, p=0.4918 | | | | |
| PLX3397+Anakinra+TBI vs. PLX3397+TBI  pre-score: p=0.7969  1dpi: p=0.9016  3dpi: p=0.9931  5dpi: p=0.8484 | PLX3397+Anakinra+TBI vs. Anakinra+TBI  pre-score: p=0.9217  1dpi: p=0.9016  3dpi: p=0.9931  5dpi: p=0.8484 | PLX3397+Anakinra+TBI vs. vehicle+TBI  pre-score: p=0.1102  1dpi: p=0.9882  3dpi: p=0.9931  5dpi: p=0.9992 | PLX3397+TBI  vs. vehicle+TBI  pre-score: p=0.7798  1dpi: p=0.8517  3dpi: p=0.9888  5dpi: p=0.8484 | Anakinra+TBI  vs. vehicle+TBI  pre-score: p=0.4223  1dpi: p=0.8517  3dpi: p=0.9888  5dpi: p=0.8484 |
| Fig. 2D | | | | |
| time*treatment: F (12, 69) = 1.008, p=0,4514  time: F (3, 69) = 5.185, p=0.0027  treatment: F (4, 23) = 1.277, p=0.3078 | | | | |
| PLX3397+Anakinra+TBI vs. PLX3397+TBI  pre-score: p=0.4077  1dpi: p=0.9861  3dpi: p=0.9906  5dpi: p=0.7489 | PLX3397+Anakinra+TBI vs. Anakinra+TBI  pre-score: p=0.4150  1dpi: p=0.9947  3dpi: p=0.6334  5dpi: p=0.2566 | PLX3397+Anakinra+TBI vs. vehicle+TBI  pre-score: p=0.9956  1dpi: p>0.9999  3dpi: p=0.8885  5dpi: p=0.9602 | PLX3397+TBI  vs. vehicle+TBI  pre-score: p=0.6452  1dpi: p=0.9948  3dpi: p=0.6419  5dpi: p=0.9847 | Anakinra+TBI  vs. vehicle+TBI  pre-score: p=0.6530  1dpi: p=0.9986  3dpi: p=0.1500  5dpi: p=0.6565 |

one-way ANOVA or Kruskal-Wallis test depending on parametric or non parametric data

p_1_: PLX3397+Anakinra+TBI vs. PLX3397+TBI

p_2_: PLX3397+Anakinra+TBI vs. Anakinra+TBI

p_3_: PLX3397+Anakinra+TBI vs. vehicle+TBI

p_4_: PLX3397+TBI vs. vehicle+TBI

p_5_: Anakinra+TBI vs. vehicle+TBI

| Fig. 2E | Fig. 2F |
| --- | --- |
| Kruskal-Wallis statistic: 13.02, p=0.0112  p_1_=0.0448  p_2_>0.9999  p_3_=0.0124  p_4_>0.9999  p_5_=0.6802 | treatment: F (4, 23) = 11.45, p<0.0001  p_1=_0.9854  p_2_=0.9854  p_3_=0.0001  p_4_=0.0001  p_5_=0.0002 |
| Fig. 2G | **Fig. 2H** |
| Kruskal-Wallis statistic: 10.63, p=0.0310  p_1_=0.9819  p_2_>0.9999  p_3_=0.0391  p_4_>0.9999  p_5_>0.9999 | Kruskal-Wallis statistic: 9.225, p=0.0557  p_1_>0.9999  p_2_>0.9999  p_3_=0.0867  p_4_=0.1740  p_5_=0.2766 |
| Fig. 2I | **Fig. 2J** |
| Kruskal-Wallis statistic: 9.019, p=0.0606  p_1_=0,0510  p_2_>0.9999  p_3_=0.7697  p_4_>0.9999  p_5_>0.9999 | treatment: F (4, 23) = 3.943, p=0.0140  p_1_=0.9800  p_2_=0.9800  p_3_=0.0274  p_4_=0.0334  p_5_=0.0501 |

**Table S5. Statistics for Fig. 3**

one-way ANOVA

p_1_: PLX3397+Anakinra+TBI vs. PLX3397+TBI

p_2_: PLX3397+Anakinra+TBI vs. Anakinra+TBI

p_3_: PLX3397+Anakinra+TBI vs. vehicle+TBI

p_4_: PLX3397+TBI vs. vehicle+TBI

p_5_: Anakinra+TBI vs. vehicle+TBI

| Fig. 3B | Fig. 3C |
| --- | --- |
| treatment: F (3, 20) = 3.771, p=0.0270  p_1_=0.6768  p_2_=0.4886  p_3_=0.2558  p_4_=0.1180  p_5_=0.0249 | treatment: F (3, 19) = 6.168, p=0.0042  p_1_=0.2609  p_2_=0.1220  p_3_=0.2491  p_4_=0.0210  p_5_=0.0047 |

**Table S6. Statistics for Fig. 4**

one-way ANOVA or Kruskal-Wallis test depending on parametric or non parametric data

p_1_: PLX3397+Anakinra+TBI vs. PLX3397+TBI

p_2_: PLX3397+Anakinra+TBI vs. Anakinra+TBI

p_3_: PLX3397+Anakinra+TBI vs. vehicle+TBI

p_4_: PLX3397+TBI vs. vehicle+TBI

p_5_: Anakinra+TBI vs. vehicle+TBI

| Fig. 4B | Fig. 4C |
| --- | --- |
| treatment: F (3, 20) = 5.431, p=0.0067  p_1_=0.0465  p_2_=0.0108  p_3_=0.0175  p_4_=0.8374  p_5_=0.8374 | treatment: F (3, 20) = 0.7389, p=0.5412  p_1_=0.7555  p_2_=0.9689  p_3_=0.9867  p_4_=0.7555  p_5_=0.9689 |
| Fig. 4D | **Fig. 4E** |
| Kruskal-Wallis statistic: 11.80, p=0.0081  p_1_=0.2421  p_2_>0.9999  p_3_=0.0049  p_4_>0.9999  p_5_=0.2110 | treatment: F (3, 20) = 0.2968, p=0.8273  p_1_=0.9538  p_2_=0.9881  p_3_=0.9881  p_4_=0.9547  p_5_=0.9881 |
| Fig. 4F | **Fig. 4G** |
| treatment: F (3, 20) = 9.200, p=0.0005  p_1_=0.0641  p_2_=0.3477  p_3_=0.0005  p_4_=0.0981  p_5_=0.0041 | treatment: F (3, 20) = 2.357, p=0.1023  p_1_=0.9986  p_2_=0.9886  p_3_=0.2070  p_4_=0.2070  p_5_=0.2070 |

**Table S7. Statistics for Fig. 7**

one-way ANOVA or Brown-Forsythe and Welch ANOVA test depending on SD varience

p_1_: PLX3397+Anakinra+TBI vs. PLX3397+TBI

p_2_: PLX3397+Anakinra+TBI vs. Anakinra+TBI

p_3_: PLX3397+Anakinra+TBI vs. vehicle+TBI

p_4_: PLX3397+TBI vs. vehicle+TBI

p_5_: Anakinra+TBI vs. vehicle+TBI

| Fig. 7B | Fig. 7C |
| --- | --- |
| treatment: F (3, 20) = 19.68, p<0.0001  p_1_=0.0293  p_2_<0.0001  p_3_<0.0001  p_4_=0.0014  p_5_=0.2285 | treatment: F (3, 20) = 0.9613, p=0.4302  p_1_=0.8110  p_2_=0.7173  p_3_=0.8854  p_4_=0.7750  p_5_=0.6323 |
| Fig. 7D | **Fig. 7F** |
| Brown-Forsythe ANOVA test:  F* (DFn, DFd) = 5.389 (3.000, 12.82), p=0.0127  p_1_=0.0499  p_2_=0.2635  p_3_=0.0056  p_4_=0.9144  p_5_=0.8967 | treatment: F (3, 20) = 10.20, p=0.0003  p_1_=0.0003  p_2_=0.0100  p_3_=0.0015  p_4_=0.6107  p_5_=0.6107 |
| Fig. 7G |  |
| treatment: F (3, 20) = 12.02, p=0.0001  p_1_=0.0156  p_2_=0.0008  p_3_=0.0001  p_4_=0.0841  p_5_=0.3433 |  |

**Table S7. Statistics for Fig. S1**

two-way ANOVA

| Fig. S1 male | | | | |
| --- | --- | --- | --- | --- |
| time*treatment: F (16, 36) = 1.895, p=0.0553  time: F (4, 36) = 8.727, p<0.0001  treatment: F (4, 9) = 2.029, p=0.1737 | | | | |
| PLX3397+Anakinra+TBI vs. PLX3397+TBI  1dpi: p=0.9842  2dpi: p=0.4981  3dpi: p=0.8846  4dpi: p=0.9615  5dpi: p=0.9642 | PLX3397+Anakinra+TBI vs. Anakinra+TBI  1dpi: p=0.9952  2dpi: p=0.8871  3dpi: p=0.8846  4dpi: p=0.7304  5dpi: p=0.9642 | PLX3397+Anakinra+TBI vs. vehicle+TBI  1dpi: p=0.9786  2dpi: p=0.8476  3dpi: p=0.0848  4dpi: p=0.9615  5dpi: p=0.4430 | PLX3397+TBI  vs. vehicle+TBI  1dpi: p=0.7179  2dpi: p=0.8871  3dpi: p=0.0218  4dpi: p=0.9615  5dpi: p=0.1750 | Anakinra+TBI  vs. vehicle+TBI  1dpi: p=0.9842  2dpi: p=0.8871  3dpi: p=0.3671  4dpi: p=0.8273  5dpi: p=0.6192 |
| Fig. S1 female | | | | |
| time*treatment: F (16, 36) = 2.263, p=0.0209  time: F (4, 36) = 7.495, p=0.0002  treatment: F (4, 9) = 0.8442, p=0.5312 | | | | |
| PLX3397+Anakinra+TBI vs. PLX3397+TBI  1dpi: p=0.8901  2dpi: p=0.8938  3dpi: p=0.9207  4dpi: p=0.6582  5dpi: p=0.9630 | PLX3397+Anakinra+TBI vs. Anakinra+TBI  1dpi: p=0.9533  2dpi: p=0.8938  3dpi: p=0.9207  4dpi: p=0.9224  5dpi: p=0.9630 | PLX3397+Anakinra+TBI vs. vehicle+TBI  1dpi: p=0.1972  2dpi: p=0.9755  3dpi: p=0.9613  4dpi: p=0.2745  5dpi: p=0.0822 | PLX3397+TBI  vs. vehicle+TBI  1dpi: p=0.6882  2dpi: p=0.9697  3dpi: p=0.9613  4dpi: p=0.9224  5dpi: p=0.1296 | Anakinra+TBI  vs. vehicle+TBI  1dpi: p=0.1773  2dpi: p=0.9697  3dpi: p=0.9613  4dpi: p=0.6424  5dpi: p=0.0526 |

**Table S8. Statistics for Fig. S2**

two-way ANOVA

| Fig. S2 male | | | | |
| --- | --- | --- | --- | --- |
| time*treatment: F (8, 46) = 4.667, p<0.0003  time: F (2, 46) = 2.146, p=0.1285  treatment: F (4, 23) = 5.190, p=0.0040 | | | | |
| PLX3397+Anakinra+TBI vs. PLX3397+TBI  1dpi: p>0.9999  3dpi: p=0.5953  5dpi: p=0.3227 | PLX3397+Anakinra+TBI vs. Anakinra+TBI  1dpi: p=0.9926  3dpi: p=0.7745  5dpi: p=0.7358 | PLX3397+Anakinra+TBI vs. vehicle+TBI  1dpi: p=0.9983  3dpi: p=0.1026  5dpi: p=0.0140 | PLX3397+TBI  vs. vehicle+TBI  1dpi: p=0.9952  3dpi: p=0.0017  5dpi: p<0.0001 | Anakinra+TBI  vs. vehicle+TBI  1dpi: p>0.9999  3dpi: p=0.6671  5dpi: p=0.2561 |
| Fig. S2 female | | | | |
| time*treatment: F (8, 46) = 3.859, p=0.015  time: F (2, 46) = 1.895, p=0.1619  treatment: F (23, 46) = 1.838, p=0.0394 | | | | |
| PLX3397+Anakinra+TBI vs. PLX3397+TBI  1dpi: p=0.7628  3dpi: p=0.9706  5dpi: p=0.8815 | PLX3397+Anakinra+TBI vs. Anakinra+TBI  1dpi: p=0.9936  3dpi: p=0.5710  5dpi: p=0.7628 | PLX3397+Anakinra+TBI vs. vehicle+TBI  1dpi: p=0.5488  3dpi: p=0.0746  5dpi: p=0.0005 | PLX3397+TBI  vs. vehicle+TBI  1dpi: p=0.9968  3dpi: p=0.2709  5dpi: p=0.0106 | Anakinra+TBI  vs. vehicle+TBI  1dpi: p=0.8005  3dpi: p=0.7820  5dpi: p=0.0213 |
